# African swine fever knowledge, attitudes and practices of pig farmers of Saint Kitts, Nevis, Saint Eustatius, Saint Lucia and Saba; West Indies

**DOI:** 10.3389/fvets.2025.1710806

**Published:** 2025-12-10

**Authors:** Luis Pablo Hervé-Claude, Christa Gallagher, Maria Jose Navarrete-Talloni, Lisa Boden, Juan Pablo Villanueva-Cabezas

**Affiliations:** 1 Department of Biomedical Sciences, Ross University School of Veterinary Medicine, Basseterre, Saint Kitts and Nevis; 2 Lewyt College of Veterinary Medicine, Long Island University, Brookville, NY, United States; 3 Royal (Dick) School of Veterinary Studies and the Roslin Institute, University of Edinburgh, Roslin, United Kingdom; 4 Department of infectious diseases, the University of Melbourne, at the Peter Doherty Institute for Infection and Immunity, Melbourne, VIC, Australia; 5 Facultad de Medicina Veterinaria, Universidad San Sebastian, Concepcion, Chile

**Keywords:** ASF, Caribbean, swine, KAP, farming, backyard, small holder

## Abstract

**Introduction:**

African Swine Fever (ASF) is an important transboundary animal disease that was re-introduced to the Americas in 2021 and is at risk of spreading through the Caribbean region. There is an active outbreak of ASF in the Dominican Republic and Haiti, and risk of onward transmission which could affect other islands, including the West Indies. If further spread of ASF occurs, pig farmers are likely to play a key role in prevention and response to an incursion of the disease. However, there is limited data about the demographics, knowledge, attitudes and practices (KAP) of pig producers in the West Indies.

**Methods:**

This study aims to describe swine production and trade within five island territories and to collect KAP data using a survey to improve pig husbandry practices and inform African Swine Fever disease control and preparedness.

**Results:**

Pig production in this region appears to be dominated by backyard, smallholder farms with active intra-island trade and important deficiencies in biosecurity. Few farmers have any knowledge on ASF, as only 29.6% (21/71) indicated to have some knowledge about ASF. Commercial feed use is widespread (95.7% of farms, 68/71), as is the feeding of household scraps (57.8%, 41/71). International legal live pig trading is limited in its frequency and volume.

**Discussion:**

The heterogeneous political, cultural and geographical nature of the West Indies means that it is important to consider each island territory individually to appreciate and consider contextual aspects of pig production for improved preparedness. Farmers in this study proved to have little knowledge on ASF, practice some high-risk behaviors (swill & scrap feeding) and lack adequate biosecurity practices to keep the region safe from ASF.

## 1 Introduction

African Swine fever (ASF) is a viral, highly contagious, notifiable disease of domestic and wild suids (1). Over the past decade, ASF has spread rapidly resulting in severe disruptions in the global swine sector (2). Although the financial impact of the current ASF panzootic is yet to be estimated, costs in 2019 for China alone were estimated between US$ 60–296.6 billion (3), with other several billion in losses due to global trade disturbances, mitigation and control measures (4). Among smallholders, the high morbidity and mortality associated with ASF outbreaks translate into socioeconomic burden and food insecurity, ultimately affecting lives and livelihoods (5).

**Figure 1:**
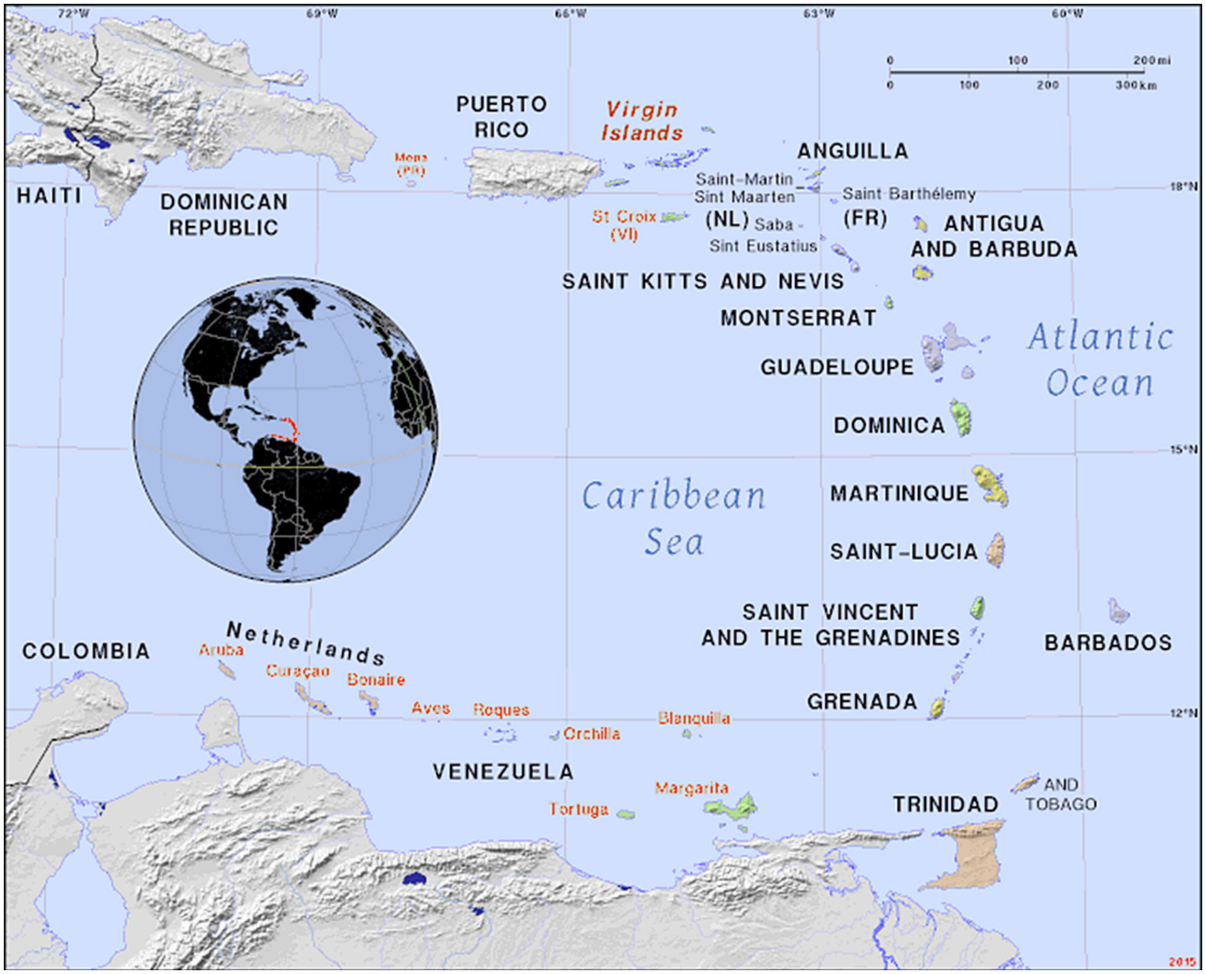
Map of the Caribbean and the West Indies. Here the islands of Saba, Saint Eustatius, Saint Kitts, Nevis and Saint Lucia can be observed. Please note on the upper left corner a partial view of Hispaniola Island (Haiti and the Dominican Republic), currently as of 2025 with an active African Swine Fever outbreak. Adapted from https://ian.macky.net/pat/map/lant/lantblu.gif. In the public domain.

In 2021, an ASF outbreak that began in the Dominican Republic and quickly spread into Haiti broke a 40-year ASF notification hiatus in the Americas (6, 7). La Hispaniola is part of the West Indies— a group of island nations and territories of varying sizes (Figure 1), political organization, languages, cultural heritages and development (8). Pigs are an integral part of the West Indies culture, with pork as a relevant protein source for many communities (9). Wild boars are commonly found in larger islands like Jamaica, Hispaniola, Trinidad and Saint Lucia, while smaller islands occasionally report the presence of escaped pigs in the wild (9).

In 2022, the Food and Agriculture Organization (FAO) conducted a qualitative risk assessment for the introduction of the African Swine Fever virus to the Caribbean and the Americas (10). Participation by small territories in the West Indies region was low for unknown reasons. Furthermore, the risk assessment highlighted that the informal trade of live pigs and pork products is widespread in the region but poorly documented, contributing to significant uncertainty in assessing ASF risks. The West Indies is characterized by limited veterinary capacity, and therefore backyard and smallholder systems, predominant in the region, often operate with minimal biosecurity and limited professional support, further increasing the risk of disease transmission. The FAO assessment underscored the urgent need for improved data collection on pig farming systems and trade networks to enhance risk assessment and inform targeted ASF prevention strategies. FAO published a guideline for ASF prevention in resource-limiting settings (11). These recommendations included “Core biosecurity measures” which are segregation measures, pig containment and cleaning and disinfection actions as key activities to prevent infection and spread of ASF.

In 2025, ASF is endemic in La Hispaniola (12), posing a significant risk for ongoing ASF transmission and spread throughout the rest of the West Indies and into the Americas, where pig production is an important economic activity (13). Understanding farmers’ level of knowledge, attitudes and practices in relation to farming and pig diseases is a key element towards establishing effective preventive measures to thwart disease incursion and spread (14, 15). Knowledge, Attitudes and Practices (KAP) surveys are simple tools that facilitate the collection of key information to plan interventions, ensuring that resources and capacity are used effectively. This study aims to address these gaps (large information gaps on husbandry practices of Caribbean pig farmers, uncertainty about their knowledge of ASF and lack of information on how they trade pig and pig products) and by systematically characterizing pig farmers across selected territories of the West Indies to facilitate assessing the implications of their practices for ASF prevention and control.

## 2 Methods

This study was approved by the ethics committee of Ross University School of Veterinary Medicine (RUSVM) (23-09-XP/HERVE) and The University of Edinburgh (HERC_2023_096). This study is reported according to the Strengthening the Reporting of Observational Studies in Epidemiology (STROBE) guidelines.

### 2.1 Study area

This study was conducted in the region known as The West Indies — a conglomerate of Islands that includes 13 independent countries and 19 dependencies/territories. These islands are linked by a network of air travel and, to a lesser extent, by intra-national and international ferry services (16). However, islands are also connected through a complex network of freight trade and fishermen. The study sample frame included pig farmers from five island territories: Nevis, Saint Kitts, Saint Lucia, Saint Eustatius (Statia), and Saba (Figure 1). The West Indian pig population is poorly characterized. Larger islands such as Jamaica, la Hispaniola and Cuba have larger and more developed pig sectors whereas in smaller territories, such as the West Indies, pig production is predominantly made of backyard and commercial farmers with small numbers of pigs.

### 2.2 Sampling frame

Participants in the study were recruited through a convenience sample of swine farmers from five island territories within the West Indies. This approach was necessary due to the absence of livestock census data (location and numbers of pigs) for the West Indies region. Participating Islands territories, therefore, were selected based on the presence of a country collaborator (usually the chief veterinary officer) willing and available to support the study. Country collaborators enrolled pig farmers based on their willingness to participate and feasibility to be interviewed.

### 2.3 Survey

A Knowledge, Attitudes and Practices (KAP) survey was developed and comprised of multiple choice and open-ended questions (see Annex 1 for a copy). The survey was designed to investigate farmer demographics and KAP in four areas: (i) Perception of ASF risk and clinical presentation, (ii) knowledge of ASF routes of spread and (iii) Awareness of biosecurity and control measures and (iv) Farm practices. Descriptive information about farming practices, including pig numbers, pig feeding, pig movements and use, pig/pork product trade, farm infrastructure, personnel on the farm and pig disease monitoring was collected.

The survey was developed in Qualtrics (Qualtrics, Provo, UT), tested by the researchers Luis Pablo Hervé Claude (LPHC), Juan Pablo Villanueva-Cabezas (JPVC) and Lisa Boden (LB) who have previously developed KAP surveys for this and other transboundary infectious diseases. Surveyors were met online or in person to discuss the study, revise the questionnaire, and clarify questions before it was applied to the farmers. Country collaborators (CC) included a local Extension/Technical officer, a Government/State Veterinarian and the Chief Veterinary Officer (CVO). These CCs agreed to administer the surveys as they knew the location of most farm premises in their territory and had a good rapport with the participants, increasing likelihood of participation and survey completion. Farmers who consented to participate read and signed an informed consent form and were interviewed at the farm or the Veterinary Services offices. The surveys were completed between February and July 2024. Data was collected in paper through in-person surveys, either in the farm premises or in the local Ministry of Agriculture Office. The average interview took approximately 25 min to be completed. Seventy-one surveys were completed among all five island territories.

### 2.4 Data analysis

Data was recorded by LPHC in *Qualtrics* for analysis. Questions 11 (Q11) (“Where do you get feed for your pigs?”) and 12 (Q12) (“Where do you buy your commercial feed”) were cross-checked to validate the type and source of pig feed. Question 11 was manually reviewed by LPHC to ensure that all farmers that indicated buying commercial feed on Q11 also indicated the source of it in Q12. Furthermore LPHC checked that all farmers that indicated not using commercial feed, indicated so in Q12 by selecting the alternative “I do not use commercial feed.” Descriptive statistics were calculated using SAS 9.4 (SAS Institute, Cary NC) and the main features of Caribbean farmer agricultural practices are presented within the manuscript in the form of frequencies, averages, median, proportions, percentages, etc and also graphically represented using radar charts/spider plots. The responses to open-ended questions were individually reviewed and subsequently classified into 3 themes (Box 1) using an inductive approach, a qualitative content analysis that involved reading responses and creating codes and themes based on those. Manual coding using MS Excel was done by LPHC. In some cases, the response rate was very low (e.g., “Do you have other names for ASF?,” with only one alternative name proposed), triggering no further evaluation. Open-ended questions included in the survey are detailed in Box 1.

**BOX 1** Open-ended questions included in the KAP survey

Farm practices

- Who monitors pig health?
- What do you do if pigs are sick?
- To whom have you sold a sick pig?
- If your pigs look lethargic, have diarrhea, and there is mortality, which husbandry practices would you change
- Do you allow visitors in your farm?

Knowledge about ASF

- What did you know about ASF?
- Where did you hear about ASF?
- Do you have other names for ASF?
- Why might high pig mortality events not be reported?
- Why would you not suspect ASF if your pig has clinical symptoms?
- Have you seen your pigs interacting with wild animals?

Awareness of response required

- What do you think is your role in preventing disease from occurring and spreading in pigs?
- What would make it easier for you to better protect your pigs from disease?
- What type of activities/measures would help pig farmers prepare for ASF? (like meetings, workshops, field visits, etc.)

## 3 Results

A total of 71 surveys were completed across the five island territories: Saint Kitts (n = 19), Nevis (n = 20), Saint Eustatius (or Statia) (n = 10), Saba (n = 3) and Saint Lucia (n = 19). As Saint Eustatius and Saba are both Dutch Overseas territories, and considering low survey numbers for Saba, descriptive statistics for these islands were combined into a single category named “Statia/Saba” (n = 13). Although this has the risk of averaging results against specific island particulars, the researchers decided to do so as the total number of farmers in Saba (n = 3), would not have allowed for any further calculations, and we wanted to be able to include their contributing data. The average farmer’s age was 48.7 (median 46, min. 21, max. 73). Across the islands, 17.9% of farmers were 60 + years old. Most farmers (approximately 60%) were educated to Secondary School level (Table 1).

**Table 1:** Demographics of pig farmers in Nevis, Statia/Saba, Saint Kitts and Saint Lucia, West Indies in 2024.

Demographics	Nevis	Saba/Statia	Saint Kitts	Saint Lucia
No. of surveys	20	13	19	19
Ave. farmer age Years (n)	43.6 (19/20)	43 (2/11)	46.1 (18/19)	57.4 (18/19)
Max. education level	Nevis	Saba/Statia	Saint Kitts	Saint Lucia
No formal education	0	0	0	5.6% (1/18)
Primary	20% (4/20)	0	0	22.2% (4/18)
Secondary	45% (9/20)	72.7% (8/11)	73.7% (14/19)	55.6% (10/18)
College	15% (3/20)	27.3% (3/11)	15.8% (3/19)	5.6% (1/18)
University	20% (4/20)	0	10.5% (2/19)	11.1% (2/18)
Pig Herd Size	Nevis	Saba/Statia	Saint Kitts	Saint Lucia
Median (IQR)	12 (30)	11 (9)	34 (28)	33.5 (109)

The median pig herd size across the island territories was 22, with various animal types represented across farms (boars, sows, piglets, growers, etc.). Farm size ranged from 3 to 399 pigs, with most farmers having less than 10 pigs (32.4%) and only 10% had 100 or more pigs.

### 3.1 Farming premises and practices

**Figure 2:**
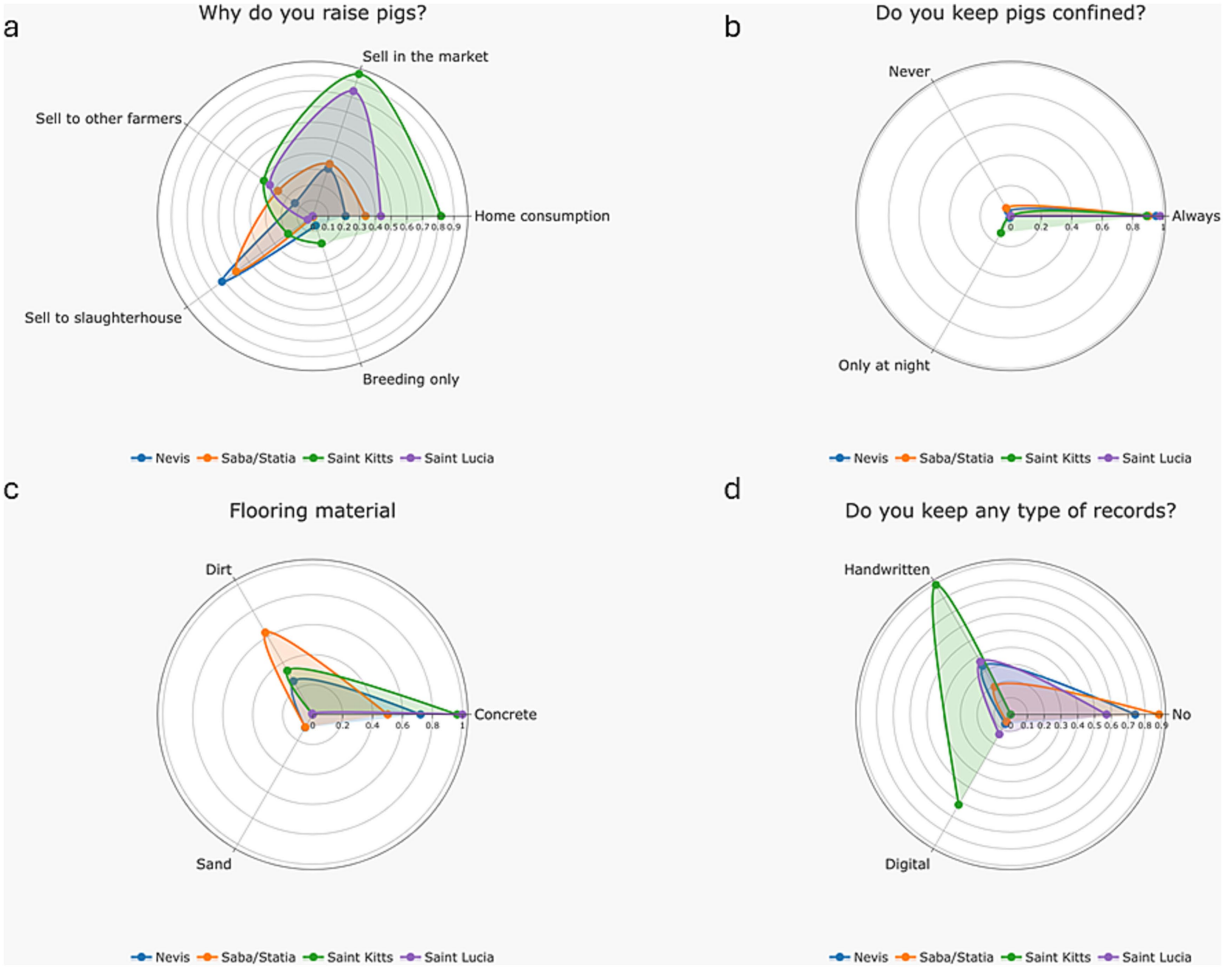
Different aspects linked with farming premises and practices, reported by farmers in Nevis, Statia, Saba, Saint Kitts and Saint Lucia in 2024; **(a)** The reason why farmers raise pigs; **(b)** The confinement type used for pigs; **(c)** The flooring material used; **(d)** Record keeping.

In the West Indies, pigs are kept for various reasons, including sale to the market (64.8%, 46/71), home consumption (46.8%, 33/71), sale to slaughterhouses (38%, 27/71) and sale to other farmers (31%, 22/71) (Figure 2a). Full-time confinement of pigs was common (94.3%, 66/70); only four farmers (5.6%, 4/70) reported night confinement only or letting pigs roam free. (Figure 2b). Pigs are predominantly kept confined on concrete floors. A small number of farms had dirt floors (28.2%, 20/71). Two farms reported sand floors (one in Nevis and one in Saba/Statia) (Figure 2c). Across islands, farms were frequently operated by the farmer plus one or two family members and/or one worker. Half of farmers (50.7%, 36/71) do not keep production records of any type; 43.6% (31/71) keep handwritten records, and 21.1% (15/71) keep digital records (Figure 2d).

**Figure 3:**
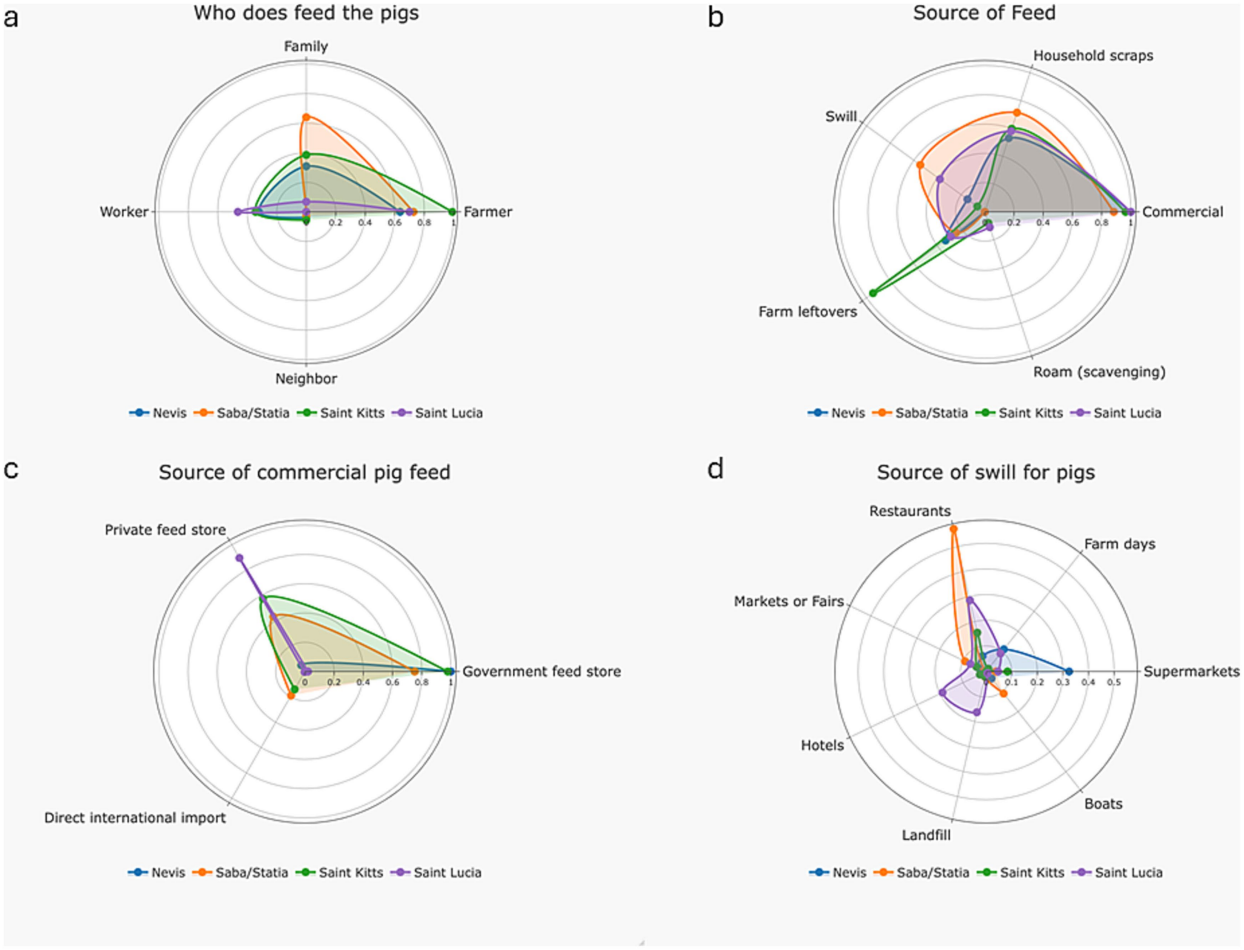
Different aspects linked with pig feed and feeding, reported by farmers in Nevis, Statia, Saba, Saint Kitts and Saint Lucia in 2024; **(a)** Person in charge of feeding pigs; **(b)** Main sources of pigs feed; **(c)** Sources of commercial pig feed; **(d)** Source of feed used for swill.

The responsibility for pig feeding is usually shared by different farm personnel. Feeding is primarily done by the farmers (77.5%, 55/71), a family member (33.8%, 24/71), or a worker (31%, 22/71) (Figure 3a). Sources of feed are described in Figure 3b. Most farmers (90.1%, 64/71) used commercial feed. Among farmers that fed commercial products, 70.4% (50/71) sourced them from government feed stores, 47.9% (34/71) from private stores, and 9.9% (7/71) used direct international shipments (Figure 3c). Over half of farmers fed their pigs household scraps (57.8%, 41/71), farm leftovers (39.4%, 28/71), and swill (25.4%, 18/71). Household scraps are the unprocessed leftovers of food in a household, farm leftovers are the remainder of crops, fruits or vegetables that are not commercialized and left in the farm and swill is post processed (heated/boiled) household scraps or farm leftovers, to create a semi-liquid feed. Some farmers allow pigs to scavenge for food (5.6%, 4/71). Produce to prepare swill was obtained from multiple sources including restaurants (22.5%, 16/71), supermarkets (14.1%, 10/71), field/farm days (7%, 5/71), hotels (7%, 5/71), markets (4%, 3/71), landfills (5.6%, 4/71) and anchored vessels at the harbor (1.4%, 1/71) Swill sources for farmers in each island territory are presented in Figure 3d.

### 3.2 Pig movement

**Figure 4:**
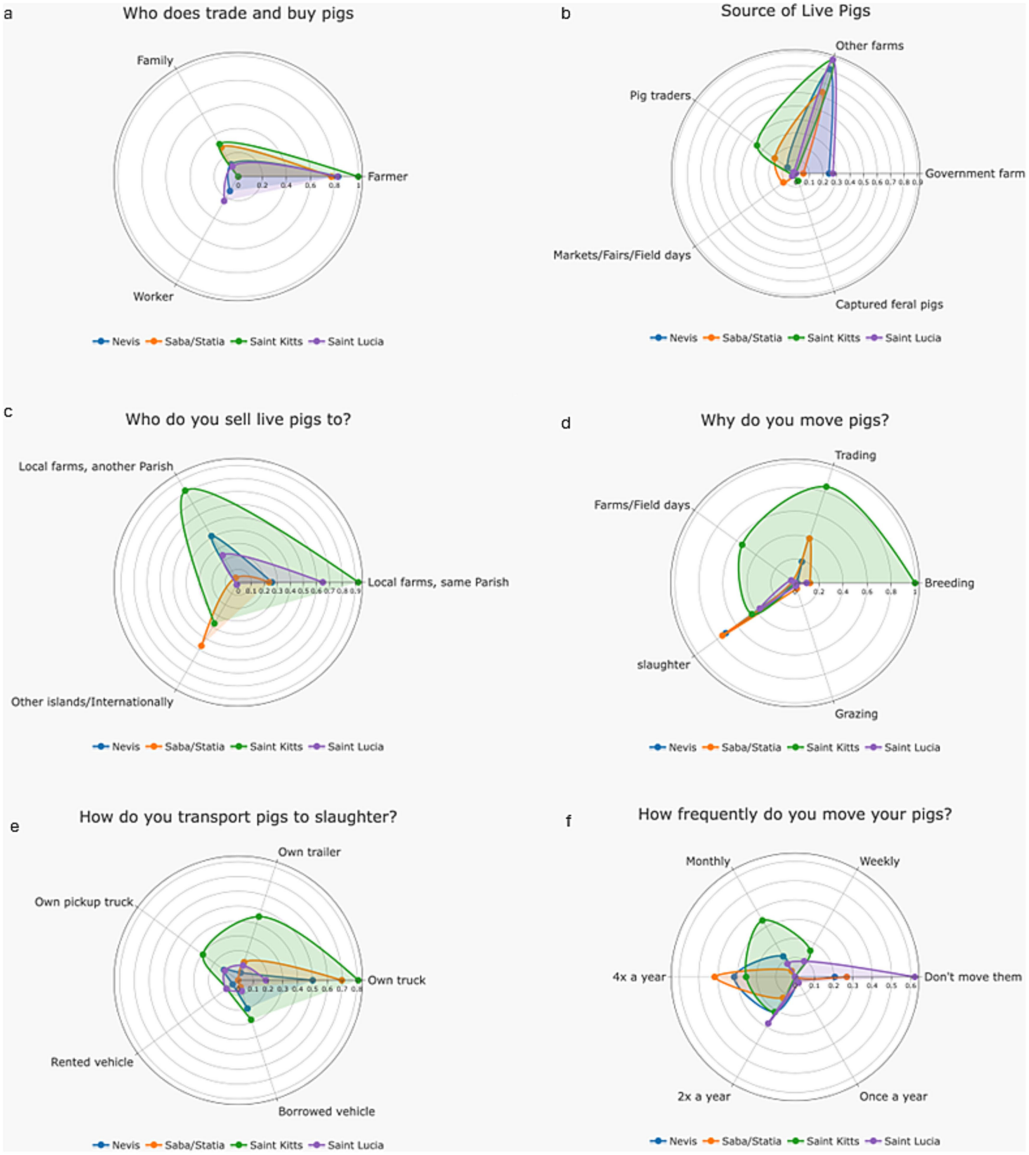
Different aspects linked with pig trade and movements, reported by farmers in Nevis, Statia, Saba, Saint Kitts and Saint Lucia in 2024; **(a)** Person in charge of pig trading; **(b)** Main source of live pigs; **(c)** Destination of sold pigs; **(d)** Reasons for moving pigs; **(e)** How pigs are transported; **(f)** How frequently are pigs moved.

Pig movements were explored to understand trading practices and potential disease spread in the region. Pig trading is conducted by farmers (85.9%, 61/71), family members (18.3%, 13/71) or workers (8.5% 6/71) (Figure 4a). New pigs are primarily obtained from other pig farms (83%, 58/70), with traders sourcing pigs to only 15.7% (11/70) of the farmers interviewed. Two farmers (2.9%, 2/70), one from Saba/Statia and one from Saint Kitts, mentioned field/farm days (agricultural events, organized by the local authorities, where farmers share knowledge, receive training, showcase their animals and crops and trade) as an alternative source for their pigs. A single farmer (1.4%, 1/70) declared to have obtained pigs captured from the wild (Figure 4b).

Most farmers (80%; 57/71) sell live pigs as part of their normal farming activities, mainly to other farms in the same Parish or area (54.9%, 39/71), more distant farms in the same island (42.3%, 30/71) or internationally/to other islands (19.7%, 14/71). This international/across island trade was restricted to Saint Kitts and Saba/Statia, with no reports of trade to other islands from Nevis or Saint Lucia (Figure 4c).

Slaughter was the most common reason for farmers to move their pigs (53.5%, 38/71), followed by trading (35.2%, 25/71), breeding (31%, 22/71) and farm/field days -sort of agricultural fairs where farmers and the general population meet—(14.1%, 10/71) (Figure 4d). Pigs are primarily moved to slaughter in farmer-owned trucks, trailers or pickup trucks (53.5%, 38/71; 16.9%, 12/71 and 14.1%, 10/71, respectively) but some borrowed (16.9%, 12/71) or rented vehicles and (4.3%, 3/71) (Figure 4e). Sharing vehicles is a widespread practice in Saint Kitts (63.2%, 12/71) but is less important on all other islands. The same phenomenon is observed for equipment sharing (St Kitts—62%, 12/19). Pig movements vary across islands (Figure 4f); however, most farmers move their animals between 2 and 4 times a year (22.5%, 16/71 for both), and a small proportion move pigs weekly (5.6%, 4/71).

### 3.3 Pig processing

**Figure 5:**
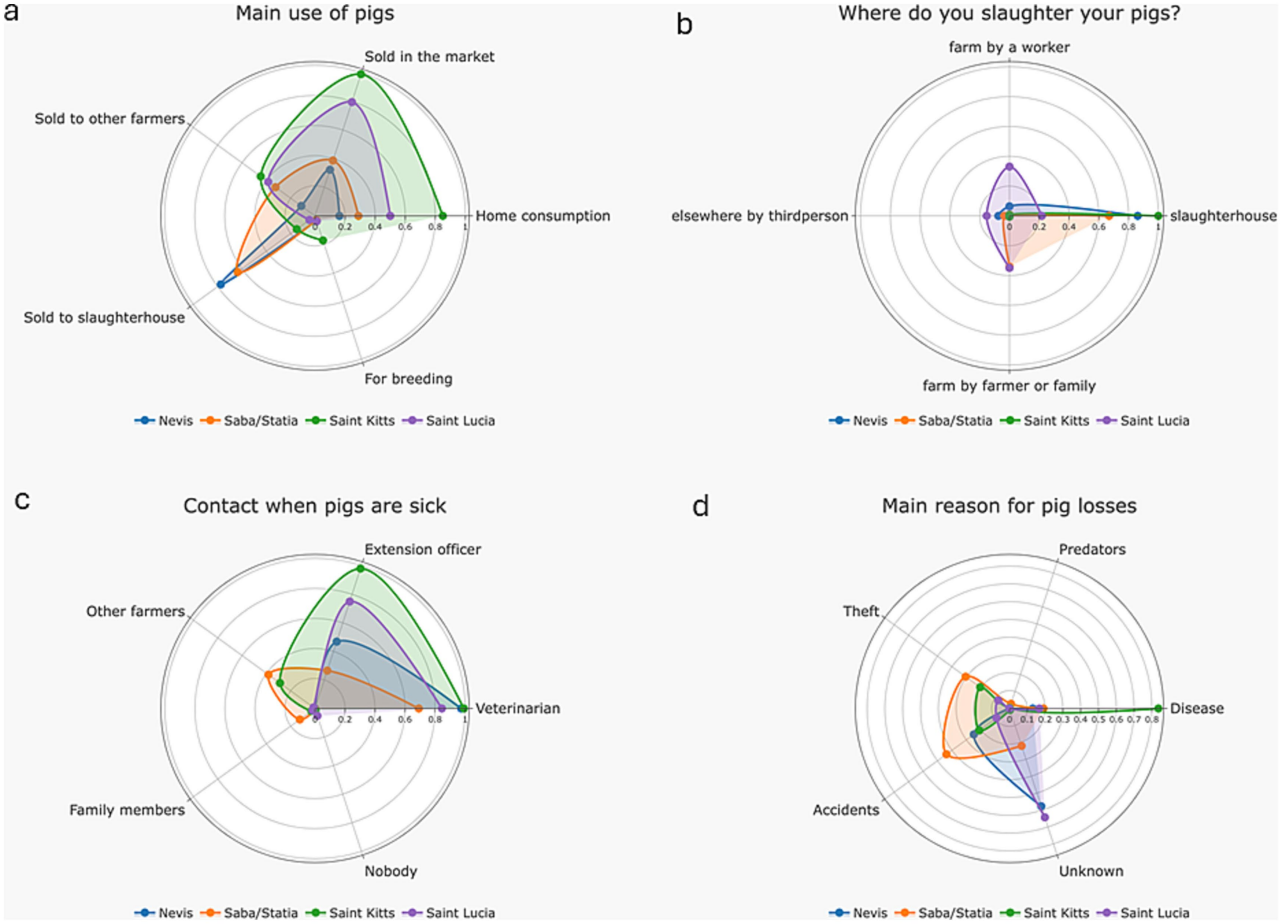
Reasons for pig rearing, slaughter and losses, as reported by farmers in Nevis, Statia, Saba, Saint Kitts and Saint Lucia in 2024; **(a)** Main use given to pigs; **(b)** Location of pig slaughter; **(c)** Where to go person in case of sick pigs; **(d)** Main reasons for pig losses and mortality.

Pigs are used for many reasons including home consumption, trading in the market and selling to slaughterhouses (Figure 5a). Pig slaughter practices varied across different islands. Farmers in Saint Kitts (100%, 19/19) and Nevis (90%, 18/20) made extensive use of the slaughtering facilities on the island. In contrast, only 26.3% (5/19) of farmers in Saint Lucia utilized slaughterhouses. Those that do not use slaughterhouses, opt instead for other methods such as local slaughter by the farmer or a family member (31.6%, 6/19), a local farm worker (31.6%, 6/19), or a third party carrying out slaughter offsite (15.8%, 3/19) (Figure 5b). Over half of the farmers (53.7%, 36/67) across the islands reported selling pig products like pork meat, salami, or sausages. The production location details of those products was not reported (whether on farm or elsewhere). Interestingly, this practice was widespread in Saint Kitts, with all farmers engaging in it (100%, 19/19), while in all other islands, around one third of farmers reported doing the same (Nevis: 33.3%, 6/18; Saba/Statia: 33.3%, 4/12; Saint Lucia: 38.9%, 7/18).

### 3.4 Pig health

Most pig health monitoring work was conducted by government officials (extension officers or government veterinarians) (50.7%, 36/71) or by farmers and their families (22.5%, 16/71). When sick pigs are identified on farms, most farmers (88.7%, 63/71) contact either a veterinarian (state or private) or the extension officer/veterinary services (64.8%, 46/71) (see Figure 5c). The leading causes of pig losses across island territories were disease (32.4%, 23/71), accidents (19.7%, 14/71), and theft (12.7%, 9/71) (Figure 5d). If farmers identify sick pigs in their herds, no one would be keen to sell a sick pig (0/71), and only one (1.4%, 1/71) would send a sick pig to the slaughterhouse.

### 3.5 Biosecurity and control measures

**Figure 6:**
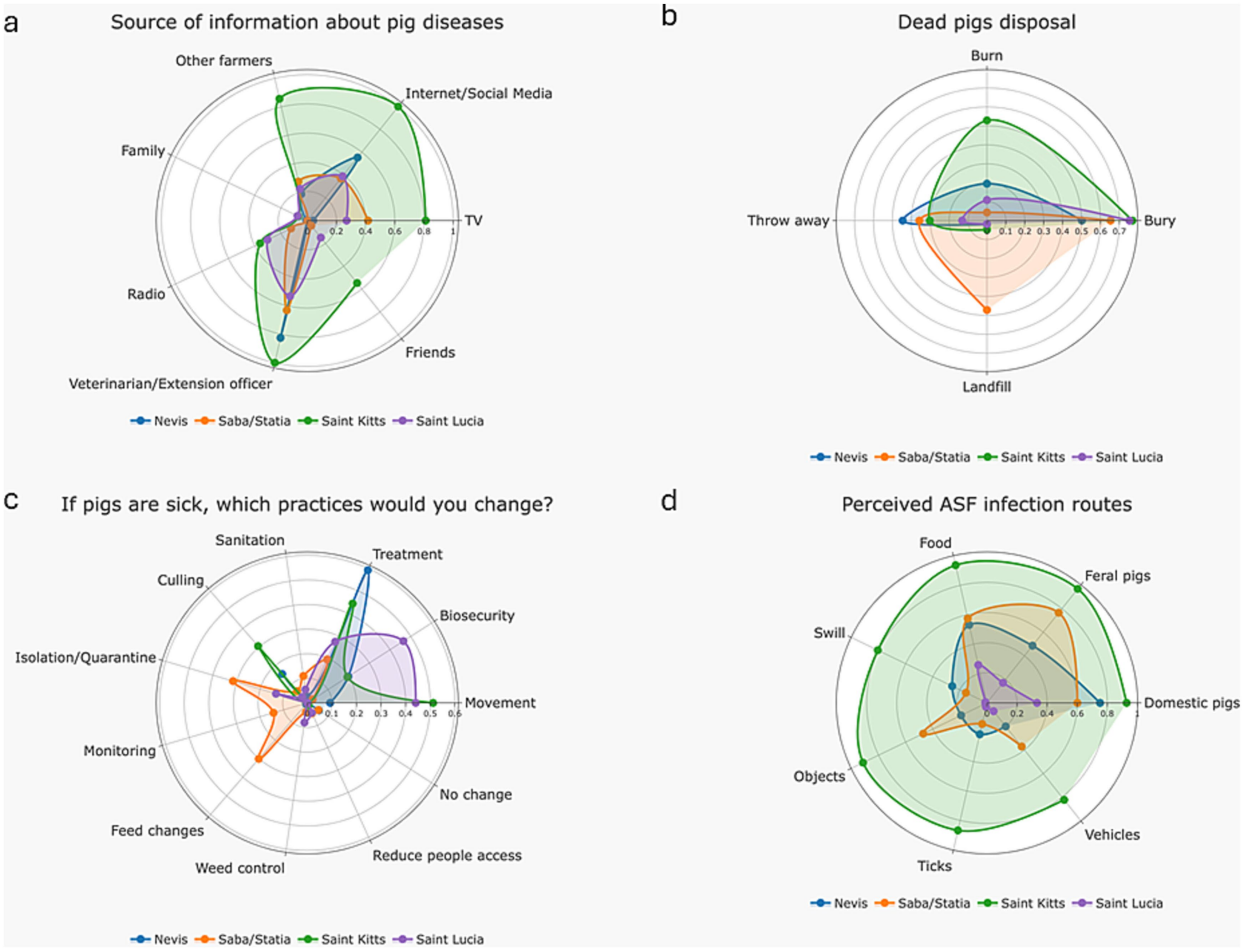
Practices when facing pig diseases, as reported by farmers in Nevis, Statia, Saba, Saint Kitts and Saint Lucia in 2024; **(a)** Main information sources on pig diseases; **(b)** Disposal methods of dead pigs; **(c)** Practice changes in the event of pigs’ diseases; **(d)** Perceived ASF infection routes.

Farmers rely on multiple information sources regarding pig diseases across the region (Figure 6a). but most information comes from the Veterinarian/Extension officer (77.5%, 55/71) and internet/social media (59.2%, 42/71). Farmers also obtained information from “Other farmers” 39.4% (28/71) and TV 38% (27/71). Friends (18.3%, 13/71), radio (16.9%, 12/71) and family (2.8%, 2/71) were also information sources.

The biggest challenge (i.e., most reported) for biosecurity was lack of money (70.4%, 50/71), lack of time (52.1%, 37/71) and lack of knowledge (49.3%, 20/71). When asked about cleaning and disinfection, 59.6% (31/52) of farmers in Nevis, Saba/Statia and Saint Lucia indicated that they disinfect their facilities, and 53.9% (28/52) specified having some form of clean and dirty areas on their farms. Further cleaning and disinfection practices are reported in Table 2. In general, biosecurity adoption is low, with important differences among islands. Disinfection of facilities (59.6%, 31/52) was prevalent, while pest control (28.9%, 15/52) was only adopted by a small number of farmers. All proposed formats for training activities were largely accepted and welcomed, including meetings, presentations, workshops and field visits. Different ways to facilitate the protection of pigs from disease were explored, the results being summarized in Table 3.

**Table 2:** Reported adoption/use of biosecurity principles in farmers from by Nevis, Statia/Saba and Saint Lucia^*^ in 2024.

Segregation	Nevis	Saba/Statia	Saint Lucia	Total
Closed Herd	30% (6/20)	23.1% (3/13)	42.1% (8/19)	32.7% (17/52)
Access Restriction	35% (7/20)	7.7% (1/13)	57.9% (11/19)	36.5% (19/52)
Clean & Dirty Areas	60% (12/20)	53.9% (7/13)	47.4% (9/19)	53.9% (28/52)
Prevent Contact	30% (6/20)	15.4% (2/13)	47.4% (9/19)	32.7% (17/52)
Pest Control	15% (3/20)	38.5% (5/13)	36.8% (7/19)	28.9% (15/52)
Cleaning & Disinfection				
Facilities	65% (13/20)	23.1% (3/13)	79% (15/19)	59.6% (31/52)
Equipment	30% (6/20)	15.4% (2/13)	68.4% (13/19)	40.4% (21/52)
Vehicles	25% (5/20)	15.4% (2/13)	47.4% (9/19)	30.1% (16/52)

* Data from Saint Kitts was not collected.

**Table 3:** What would make it easier for you to better protect your pigs from disease? Answers from West Indies farmers in 2024.

Theme	Needs	% (n)
Increased support	Training	33.1% (8/24)
	Money	33.1% (8/24)
	Resources like better feed, knowledge	29.2% (7/24)
	Government support (assistance, subsidies, interventions)	25.0% (6/24)
	Time	8.3% (2/24)
	Land ownership	4.2% (1/24)
Improved farm management	Better cleaning and disinfection	47.6% (10/21)
	Better segregation	23.8% (2/21)
	Improved animal monitoring	19.0% (4/21)
	Upgrade pig welfare	4.8% (1/21)
	Vaccination	4.8% (1/21)
Veterinary services	Increased calls/visits from the veterinarian	71.4% (10/14)
	Access to medication	14.3% (2/14)
	Access to medications from officers & response from calls	7.1% (1/14)
	More information and access to medicine	7.1% (1/14)
Improved farm facilities	Better infrastructure (piggery, shelter, repairs, upgrades)	71.4% (5/17)
	Better infrastructure for biosecurity	14.3% (1/7)
	Better equipment	14.3% (1/7)

Reported are several farmers that had answers on each linked to a theme. Some farmers indicated more than one need within a theme.

Most farmers reported that they did not receive visitors on their farms, (77.5%, 55/71). Of the farms that did, most visitors were veterinarians or extension officers (11.3%, 8/71), with some farms receiving regular visits from “*many people, buyers*” (Pers. Comm., participant #65). A few farmers reported the imposition of biosecurity measures to outside visitors, including washing boots (2.8%, 2/71). Only one farmer (1/71) requested that visitors use a footbath, cleanse and disinfect (C&D) vehicles or wear clean clothes.

Most farmers threw away deceased pigs (68.6%, 48/71). Alternatively, they buried deceased pigs (67.1%, 47/71) near the farm perimeter. Farmers (mostly in St. Kitts) also reported burning pigs (22.9%, 16/71). In Statia/Saba, farmers sent dead pigs to landfill (8.6%, 6/71) (Figure 6b).

### 3.6 Feral pigs

One third of farmers thought feral pigs were a disease concern for pig farming and most farmers believed that their pigs were not in contact with feral pigs. This belief varied between islands (St Kitts 73.7%; 14/19—St Lucia 94.7%; 18/19). In Saint Kitts a few farmers (5/19) thought their pigs “sometimes” encountered feral pigs.

### 3.7 Awareness and knowledge of African swine fever

Awareness of ASF amongst participants was low (29.6%, 21/71 overall), with variability between islands (14.3% (3/19) of farmers in St. Lucia, 19.1% (4/20) in Nevis, 33.3% (4/13) from Saba/Statia and 33.3% (6/19) in Saint Kitts). Only 18 (25.4%) farmers were aware that ASF outbreaks were occurring elsewhere in the world and fewer 9.9% (7/71) farmers identified the Dominican Republic/Haiti (Hispaniola) as an ASF positive island.

Clinical signs, such as lethargy, diarrhea and mortalities, would prompt farmers to treat pigs (38%, 27/71), move pigs within the farm (28.2%, 20/71), increase biosecurity (22.5%, 16/71), cull pigs (11.1%, 10/71), isolate and quarantine animals (9.9%, 7/71), improve sanitation (4.2%, 3/71) and/or change feed (5.6%, 4/71) (Figure 6c). Reporting high mortality events could not be assessed (70/71 did not respond to the question, 1/71 reported that they would not contact a veterinarian because of concerns that their pigs would be culled). Lack of knowledge about ASF (52.1%, 37/71), lack of communication from the authorities about ASF (18.3%, 13/71) and the absence of ASF on the islands (16.9%, 12/71) were offered as reasons for farmers’ behaviors. Few farmers (8.5%, 6/71) indicated they were well informed about ASF transmission routes (see Figure 6d which describes knowledge about different transmission pathways). When exploring the potential formats on which farmers would like to receive information on ASF, Workshops was the preferred option with 70.2% (47/67) of farmers selecting this option, followed by presentations 59.7% (40/67), meetings & field visits, both with 38/67 (56.7%). Four farmers did not answer this question 2.8% (4/71).

## 4 Discussion

Strengthening biosecurity measures, raising farmer awareness, and implementing robust surveillance programs are vital steps in mitigating the threat of ASF in the Caribbean (10). However, the cultural, political, and geographical diversity and varying connectivity present substantial challenges for coordinated preparedness and response efforts throughout the region (2, 17). Although the Caribbean Agricultural Health and Food Safety Agency (CAHFSA) is dedicated to collating and aggregating agricultural information to standardize animal health legislation for the region (18), there are still important challenges like the differences in legislation and differences in bodies required to enforce such legislation throughout the region (Dr. Nneka Hull-James, CAHFSA, pers. comm.). This study presents novel insight into the pig production practices of five West Indies territories.

It highlighted the relative importance of intensive intra-island pig trading compared to lower-intensity inter-island pig movements which could contribute to the risk of onward transmission of ASF should a disease incursion occur. The risk may be compounded by the relatively basic farm infrastructure, low adoption of biosecurity practices, and limited knowledge on ASF as an imminent threat for this region. Husbandry practices differ from island to island, with significant differences in feeding, slaughtering and other practices illustrating a potential risk communication and governance gap. Farmers obtain information about pigs and pigs’ diseases from radio programs, the internet/social media or through in-person interactions, with marked differences between island territories.

The nature of the pig industry in the West Indies (i.e., mainly informal and backyard pig farms) is comparative to that in ASF-affected Dominican Republic & Haiti (6, 19) and Martinique (20) and paucity of data, particularly on wild/feral pigs, presents a real challenge for islands and territories (12, 21, 22). This study managed to unveil some of the complexities of pig farming and the risk linked with potential ASF spread in the West Indies. Inter-island trade in live pigs and animal feed are important potential transmission pathways. In the West Indies, live pigs are moved to enhance genetic stock or replenish stocks for seasonal backyard producers with relatively low frequency and volume. There is a live pig trade route from Statia to Saba, which connects with another trade route between Saint Kitts & Nevis and Statia (10). These examples highlight the existence of small-scale live pig trade networks in the region that would allow for inter-island ocasional (and sometimes seasonal) possibility of ASF disease spread. Furthermore, in the case of an incursion of ASF to this region, this new information points towards a rapid intra-island spread (when facing low biosecurity and low technification pig production systems), although this remains to be further explored.

Commercial pig feed is used throughout the West Indies and is traded internationally between small island territories, posing a potential biosecurity risk. Swill feeding is also widespread in the American continent (23) and in the Dominican Republic (12). In Saint Kitts & Nevis and Saint Lucia, direct feeding of kitchen, restaurant, industrial and agricultural scraps is also widespread. These examples highlight that feed remains a major threat for disease spread in certain territories and suggests there are opportunities to raise awareness of the risks of ASF associated with feeding organic matter and table scraps to pigs.

Farmers have applied some of FAO’s Core Biosecurity Measures (11) including prevention of contact between pigs and contaminated objects through housing or fencing. Free-roaming pigs are uncommon, and containment is preferred among farmers. However, biosecurity practices, including segregation, cleaning and disinfection, pest control, restricting access and sharing of vehicles, equipment, machinery, and persons to the premises, were poorly and inconsistently applied. The on-farm slaughtering of pigs has been previously described as a high-risk action for ASF spread (24). Our results indicated that such practices are widespread in multiple territories, mainly St. Lucia, Saba and Statia. The uncontrolled slaughter increases the ASF spread risk by facilitating the release of untreated by products and residues from slaughter into the environment, where domestic and wild animals can have easy access. Sometimes by-products are purposely fed to dogs, but that behavior was not explored in the study nor observed in the field. It is hypothesized that on farm slaughter could be facilitated by cultural reasons, lack of formal slaughtering facilities or difficult access to those facilities. These findings support that biosecurity remains a major challenge, and could be improved on most farms on these territories. Adding biosecurity and the strengthening of already practiced biosecurity measures should be encouraged, such as improving confinement methods (i.e., the use of quarantine, better fences and cleaning & disinfection), highlighting the importance of the use of commercial feed to avoid feeding other riskier foods and the use of designated clothing to work with animals (a low cost but high impact biosecurity measure).

The FAO Risk Assessment highlighted a lack of information from many territories in the West Indies. This study contributed to reducing this gap, adding some information on international live pig trade routes and providing information on the intra-island pig husbandry knowledge, attitudes, and practices of pig farmers. The full network of connections among island territories in the West Indies needs to be further characterized with a deeper understanding of risk practices in the region.

This study is far from a comprehensive exploration of the West Indies, as only five islands / territories were addressed, through a limited sample of farmers recruited from a convenience sample. Even though a convenience sample has the potential of producing biased results as any non-randomized design, the lack of any sample frame or knowledge on farm locations, rendered it the best option by the researchers. Even so, this study represents an important piece of information and a cornerstone for further understanding of this usually overlooked region, closely connected to the Americas and Europe. To date, Caribbean ASF studies have focused on the Hispaniola outbreak (6, 12) or on the potential risk of spread in the region (11, 25), without addressing the intrinsic characteristics of the different island/territories, as if those were uniform specs of land on a map. The presence or not of feral/wild pigs, the occurrence of pig farms, the density of pigs and farms, the level of industrialization, the feed chain structures, the feeding practices (swill/no swill), the access to farms and restaurants food scraps, to name a few, are key characteristics that we found vary largely among island territories. If those differences are ignored, there is a risk of regional interventions that, although represent average realities, will not be applicable to these diverse island territories. The use of self-reported practices, although practical, may introduce different types of bias including recall or social desirability bias. These biases might have influenced responses to sensitive questions that explored feeding practices (swill, scraps, etc.) or farmers’ behaviors linked to sick pigs (selling, slaughtering, etc.). Finally, the sole use of descriptive analysis and lack of statistical testing was considered appropriate as the goal of this study was to describe the local situation within a diverse island ecosystem, rather than to employ analytical tests to find statistical differences that may hold little or no real meaning. The value of these results is on highlighting—to the best of our knowledge—the differences of these territories.

## Recommendations and conclusions

Data-driven decision-making should be possible in the West Indies. The close connectedness of Governmental Veterinary Services to farmers and the relatively small number of farms should facilitate easier information exchange. This is because on many of the smaller island territories Government Veterinarians are deeply connected in their small communities. To do so, the creation of farms (and farmers) databases would be an excellent starting point, along with documentation on the areas where wild pigs are found. Veterinary Extension Officers usually have a good idea of where the farms are located. With the use of mobile phones, geo localization is easy and open ware resources exist that would allow for simple maps—and identification of the high-density areas. The knowledge of the presence or absence of feral/wild pigs is available upon consultation of key on-island personnel, but usually not published. This would be the beginning of a reliable framework on which to build a more complex surveillance and management/support system. This would allow for the identification of the farms and areas at higher risk. Farmers seem to be eager for training; therefore, farmer workshops and information-sharing sessions (meetings, presentations and field visits) should be encouraged as a way of passing knowledge to farmers. As part of this, the creation of in-farm records should be emphasized. Highlighting procedural changes that require low initial investment, like the establishment of on-farm clean and dirty areas, access restrictions for people, vehicles, and animals and the correct use of cleaning and disinfection should be encouraged in this low-income resource-scarce environment. Based on our study incorporating five island territories in the West Indies it seems like farmers are ill-prepared for a potential ASF outbreak. Until now, geographical and political isolation has proven to be protective against further ASF spread, but once and *if* it arrives, the same geopolitical complexity will play against any coordinated effort to control and eliminate ASF from the region.

## Data Availability

The raw data supporting the conclusions of this article will be made available by the authors, without undue reservation.
