# Supplementary material for: African swine fever knowledge, attitudes and practices of pig farmers of Saint Kitts, Nevis, Saint Eustatius, Saint Lucia and Saba; West Indies
Source: Front Vet Sci. 2025 Dec 10;12:1710806. doi: 10.3389/fvets.2025.1710806 (PMC12729110; doi:10.3389/fvets.2025.1710806)
Supplement: Supplementary file 1 [file Table_1.docx]

Annex

**TABLE 1** Island countries and territories in the West Indies

| Administration |  | Countries / Territories |
| --- | --- | --- |
| Countries | Independent countries | Antigua and Barbuda, Bahamas, Barbados, Cuba, Dominica, Dominican Republic, Grenada, Haiti, Jamaica, **Saint Kitts*** and **Nevis***, **Saint Lucia***, Saint Vincent and the Grenadines, Trinidad and Tobago. |
| Dependencies | France | Guadeloupe, Martinique, Saint Barthélemy,  Saint Martin. |
|  | Netherlands | Aruba, Bonaire, Curaçao, **Sint Eustatius***, Sint Maarten, **Saba***. |
|  | United Kingdom | Anguilla, British Virgin Islands, Cayman Islands,  Montserrat, Turks and Caicos Islands. |
|  | United States of America | US Virgin Islands, Puerto Rico  Haiti- Navassa Island (Claimed by the United States and Haiti). |

*Territories included in our study
