# Supplementary material for: African swine fever knowledge, attitudes and practices of pig farmers of Saint Kitts, Nevis, Saint Eustatius, Saint Lucia and Saba; West Indies
Source: Front Vet Sci. 2025 Dec 10;12:1710806. doi: 10.3389/fvets.2025.1710806 (PMC12729110; doi:10.3389/fvets.2025.1710806)
Supplement: Supplementary file 2 [file Table_2.docx]

| **Q1_Location** | **Frequency** | **Percent** |
| --- | --- | --- |
| **Nevis** | 20 | 28.17 |
| **Saba** | 3 | 4.23 |
| **Saint Kitts** | 19 | 26.76 |
| **Saint Lucia** | 19 | 26.76 |
| **Statia** | 10 | 14.08 |

Q2

| **Variable** | **Label** | **N** | **Mean** | **Median** | **Lower Quartile** | **Upper Quartile** | **Quartile Range** | **Std Dev** | **Minimum** | **Maximum** |
| --- | --- | --- | --- | --- | --- | --- | --- | --- | --- | --- |
| Q2_Age  Q4_Herd_size  Q7_Family  Q8_Workers | Q2_Age  Q4_Herd_size  Q7_Family  Q8_Workers | 57  69  65  65 | 48.7  43.0  1.3  1.1 | 46.0  22.0  1.0  1.0 | 38.0  10.0  0  0 | 59.0  44.0  2.0  1.0 | 21.0  34.0  2.0  1.0 | 14.4  65.9  1.2  1.5 | 21.0  3.0  0  0 | 73.0  399.0  5.0  9.0 |

| **Q3_Education** | **Frequency** | **Percent** |
| --- | --- | --- |
| **Frequency Missing = 3** | | |
| **1 None** | 1 | 1.47 |
| **2 Primary school** | 8 | 11.76 |
| **3 Secondary school** | 41 | 60.29 |
| **4 College** | 10 | 14.71 |
| **5 University** | 8 | 11.76 |

Q4

| **Variable** | **Label** | **N** | **Mean** | **Median** | **Lower Quartile** | **Upper Quartile** | **Quartile Range** | **Std Dev** | **Minimum** | **Maximum** |
| --- | --- | --- | --- | --- | --- | --- | --- | --- | --- | --- |
| Q2_Age  Q4_Herd_size  Q7_Family  Q8_Workers | Q2_Age  Q4_Herd_size  Q7_Family  Q8_Workers | 57  69  65  65 | 48.7  43.0  1.3  1.1 | 46.0  22.0  1.0  1.0 | 38.0  10.0  0  0 | 59.0  44.0  2.0  1.0 | 21.0  34.0  2.0  1.0 | 14.4  65.9  1.2  1.5 | 21.0  3.0  0  0 | 73.0  399.0  5.0  9.0 |

Q5

| **Variable** | **Label** | **N** | **Mean** | **Median** | **Lower Quartile** | **Upper Quartile** | **Quartile Range** | **Std Dev** | **Minimum** | **Maximum** |
| --- | --- | --- | --- | --- | --- | --- | --- | --- | --- | --- |
| Q2_Age  Q4_Herd_size  Q5_1_Sows  Q5_2_Weaners  Q5_3_Boars  Q5_4_Piglets  Q5_6_Gilts  Q5_7_Growers  Q7_Family  Q8_Workers | Q2_Age  Q4_Herd_size  Q5_1_Sows  Q5_2_Weaners  Q5_3_Boars  Q5_4_Piglets  Q5_6_Gilts  Q5_7_Growers  Q7_Family  Q8_Workers | 57  69  68  53  64  56  41  17  65  65 | 48.7  43.0  8.8  21.9  2.1  16.9  2.3  1.2  1.3  1.1 | 46.0  22.0  4.0  12.0  2.0  10.0  0.0  0.0  1.0  1.0 | 38.0  10.0  3.0  6.0  1.0  6.0  0.0  0.0  0.0  0.0 | 59.0  44.0  8.0  20.0  2.0  23.0  1.0  0.0  2.0  1.0 | 21.0  34.0  5.0  14.0  1.0  17.0  1.0  0.0  2.0  1.0 | 14.4  65.9  17.1  33.4  2.0  21.3  5.5  2.8  1.2  1.5 | 21.0  3.0  1.0  0.0  0.0  0.0  0.0  0.0  0.0  0.0 | 73.0  399.0  105.0  157.0  12.0  120.0  30.0  10.0  5.0  9.0 |

| **Q6_1_govmt_farm** | **Frequency** | **Percent** |
| --- | --- | --- |
| **Frequency Missing = 3** | | |
| **No** | 57 | 83.82 |
| **Yes** | 11 | 16.18 |

| **Q6_2_other_farms** | **Frequency** | **Percent** |
| --- | --- | --- |
| **Frequency Missing = 1** | | |
| **No** | 12 | 17.14 |
| **Yes** | 58 | 82.86 |

| **Q6_3_traders** | **Frequency** | **Percent** |
| --- | --- | --- |
| **Frequency Missing = 1** | | |
| **No** | 59 | 84.29 |
| **Yes** | 11 | 15.71 |

| **Q6_4_markets_fairs_fielddays** | **Frequency** | **Percent** |
| --- | --- | --- |
| **Frequency Missing = 1** | | |
| **No** | 68 | 97.14 |
| **Yes** | 2 | 2.86 |

| **Q6_5_feral_pigs** | **Frequency** | **Percent** |
| --- | --- | --- |
| **Frequency Missing = 1** | | |
| **No** | 69 | 98.57 |
| **Yes** | 1 | 1.43 |

Q7

| **Variable** | **Label** | **N** | **Mean** | **Median** | **Lower Quartile** | **Upper Quartile** | **Quartile Range** | **Std Dev** | **Minimum** | **Maximum** |
| --- | --- | --- | --- | --- | --- | --- | --- | --- | --- | --- |
| Q2_Age  Q4_Herd_size  Q7_Family  Q8_Workers | Q2_Age  Q4_Herd_size  Q7_Family  Q8_Workers | 57  69  65  65 | 48.7  43.0  1.3  1.1 | 46.0  22.0  1.0  1.0 | 38.0  10.0  0  0 | 59.0  44.0  2.0  1.0 | 21.0  34.0  2.0  1.0 | 14.4  65.9  1.2  1.5 | 21.0  3.0  0  0 | 73.0  399.0  5.0  9.0 |

Q8

| **Variable** | **Label** | **N** | **Mean** | **Median** | **Lower Quartile** | **Upper Quartile** | **Quartile Range** | **Std Dev** | **Minimum** | **Maximum** |
| --- | --- | --- | --- | --- | --- | --- | --- | --- | --- | --- |
| Q2_Age  Q4_Herd_size  Q7_Family  Q8_Workers | Q2_Age  Q4_Herd_size  Q7_Family  Q8_Workers | 57  69  65  65 | 48.7  43.0  1.3  1.1 | 46.0  22.0  1.0  1.0 | 38.0  10.0  0  0 | 59.0  44.0  2.0  1.0 | 21.0  34.0  2.0  1.0 | 14.4  65.9  1.2  1.5 | 21.0  3.0  0  0 | 73.0  399.0  5.0  9.0 |

| **Q9_1_farmer_feeds** | **Frequency** | **Percent** |
| --- | --- | --- |
| **No** | 16 | 22.54 |
| **Yes** | 55 | 77.46 |

| **Q9_2_family_feeds** | **Frequency** | **Percent** |
| --- | --- | --- |
| **No** | 47 | 66.20 |
| **Yes** | 24 | 33.80 |

| **Q9_3_worker_feeds** | **Frequency** | **Percent** |
| --- | --- | --- |
| **No** | 49 | 69.01 |
| **Yes** | 22 | 30.99 |

| **Q9_4_neighboor_feeds** | **Frequency** | **Percent** |
| --- | --- | --- |
| **No** | 70 | 98.59 |
| **Yes** | 1 | 1.41 |

| **Q10_1_farmer_trades** | **Frequency** | **Percent** |
| --- | --- | --- |
| **No** | 9 | 12.68 |
| **Yes** | 61 | 85.92 |
| **na** | 1 | 1.41 |

| **Q10_2_family_trades** | **Frequency** | **Percent** |
| --- | --- | --- |
| **No** | 57 | 80.28 |
| **Yes** | 13 | 18.31 |
| **na** | 1 | 1.41 |

| **Q10_3_worker_trades** | **Frequency** | **Percent** |
| --- | --- | --- |
| **No** | 64 | 90.14 |
| **Yes** | 6 | 8.45 |
| **na** | 1 | 1.41 |

| **Q11_2_house_feed** | **Frequency** | **Percent** |
| --- | --- | --- |
| **No** | 30 | 42.25 |
| **Yes** | 41 | 57.75 |

| **Q11_3_swill_feed** | **Frequency** | **Percent** |
| --- | --- | --- |
| **No** | 53 | 74.65 |
| **Yes** | 18 | 25.35 |

| **Q11_4_farms_feed** | **Frequency** | **Percent** |
| --- | --- | --- |
| **No** | 43 | 60.56 |
| **Yes** | 28 | 39.44 |

| **Q11_5_roam_feed** | **Frequency** | **Percent** |
| --- | --- | --- |
| **No** | 67 | 94.37 |
| **Yes** | 4 | 5.63 |

| **Q12_1_Govmt_feed** | **Frequency** | **Percent** |
| --- | --- | --- |
| **Government Store** | 50 | 70.42 |
| **No** | 21 | 29.58 |

| **Q12_2_Private_feed** | **Frequency** | **Percent** |
| --- | --- | --- |
| **No** | 37 | 52.11 |
| **Private Stores** | 34 | 47.89 |

| **Q12_3_Intl_feed** | **Frequency** | **Percent** |
| --- | --- | --- |
| **Abroad - Private import** | 7 | 9.86 |
| **No** | 64 | 90.14 |

| **Q12_4_no_feed** | **Frequency** | **Percent** |
| --- | --- | --- |
| **I don't use commercial feed** | 1 | 1.41 |
| **No** | 70 | 98.59 |

| **Q13_1_Use_Swill** | **Frequency** | **Percent** |
| --- | --- | --- |
| **I don't use swill** | 45 | 63.38 |
| **Yes** | 26 | 36.62 |

| **Q13_2_supermarket_Swill** | **Frequency** | **Percent** |
| --- | --- | --- |
| **No** | 61 | 85.92 |
| **Supermarkets** | 10 | 14.08 |

| **Q13_3_farm_days_Swill** | **Frequency** | **Percent** |
| --- | --- | --- |
| **Farm days** | 5 | 7.04 |
| **No** | 66 | 92.96 |

| **Q13_4_restaurant_Swill** | **Frequency** | **Percent** |
| --- | --- | --- |
| **No** | 55 | 77.46 |
| **Restaurants** | 16 | 22.54 |

| **Q13_5_markets_Swill** | **Frequency** | **Percent** |
| --- | --- | --- |
| **Markets or fairs** | 3 | 4.23 |
| **No** | 68 | 95.77 |

| **Q13_7_landfill_Swill** | **Frequency** | **Percent** |
| --- | --- | --- |
| **Landfill** | 4 | 5.63 |
| **No** | 67 | 94.37 |

| **Q13_8_boats_Swill** | **Frequency** | **Percent** |
| --- | --- | --- |
| **Boats** | 1 | 1.41 |
| **No** | 70 | 98.59 |

| **Q14_live_Sell** | **Frequency** | **Percent** |
| --- | --- | --- |
| **No** | 14 | 19.72 |
| **Yes** | 57 | 80.28 |

| **Q15_1_sell_local** | **Frequency** | **Percent** |
| --- | --- | --- |
| **No** | 28 | 39.44 |
| **Other farms in your Parish** | 39 | 54.93 |
| **na** | 4 | 5.63 |

| **Q15_2_sell_island** | **Frequency** | **Percent** |
| --- | --- | --- |
| **No** | 37 | 52.11 |
| **Other farms in other Parishes** | 30 | 42.25 |
| **na** | 4 | 5.63 |

| **Q15_3_sell_abroad** | **Frequency** | **Percent** |
| --- | --- | --- |
| **No** | 53 | 74.65 |
| **Other islands** | 14 | 19.72 |
| **na** | 4 | 5.63 |

| **Q15_4_dont_sell** | **Frequency** | **Percent** |
| --- | --- | --- |
| **I don't sell live pigs** | 16 | 22.54 |
| **No** | 51 | 71.83 |
| **na** | 4 | 5.63 |

| **Q16_sell_products** | **Frequency** | **Percent** |
| --- | --- | --- |
| **Frequency Missing = 4** | | |
| **No** | 31 | 46.27 |
| **Yes** | 36 | 53.73 |

| **Q17_1_slaughterhouse** | **Frequency** | **Percent** |
| --- | --- | --- |
| **At the slaughterhouse** | 51 | 71.83 |
| **No** | 19 | 26.76 |
| **na** | 1 | 1.41 |

| **Q17_2_locally_worker** | **Frequency** | **Percent** |
| --- | --- | --- |
| **It is done in my farm/house by a worker** | 7 | 9.86 |
| **No** | 63 | 88.73 |
| **na** | 1 | 1.41 |

| **Q17_3_external_slaughter** | **Frequency** | **Percent** |
| --- | --- | --- |
| **A third person will slaughter somewhere else** | 4 | 5.63 |
| **No** | 66 | 92.96 |
| **na** | 1 | 1.41 |

| **Q17_4_me_family_local_slaughter** | **Frequency** | **Percent** |
| --- | --- | --- |
| **Myself or a family member in my farm/house** | 10 | 14.08 |
| **No** | 60 | 84.51 |
| **na** | 1 | 1.41 |

| **Q18_1_own_truck** | **Frequency** | **Percent** |
| --- | --- | --- |
| **No** | 19 | 26.76 |
| **Own truck** | 38 | 53.52 |
| **na** | 14 | 19.72 |

| **Q18_2_own_trailer** | **Frequency** | **Percent** |
| --- | --- | --- |
| **No** | 45 | 63.38 |
| **Own trailer** | 12 | 16.90 |
| **na** | 14 | 19.72 |

| **Q18_3_own_pickup** | **Frequency** | **Percent** |
| --- | --- | --- |
| **No** | 47 | 66.20 |
| **Own pickup truck** | 10 | 14.08 |
| **na** | 14 | 19.72 |

| **Q18_4_rented** | **Frequency** | **Percent** |
| --- | --- | --- |
| **No** | 54 | 76.06 |
| **Rented vehicle** | 3 | 4.23 |
| **na** | 14 | 19.72 |

| **Q18_5_borrowed_truck** | **Frequency** | **Percent** |
| --- | --- | --- |
| **Borrowed vehicle** | 12 | 16.90 |
| **No** | 45 | 63.38 |
| **na** | 14 | 19.72 |

| **Q18_6_na_truck** | **Frequency** | **Percent** |
| --- | --- | --- |
| **No** | 57 | 80.28 |
| **na** | 14 | 19.72 |

| **Q19_1_for_breeding** | **Frequency** | **Percent** |
| --- | --- | --- |
| **Breeding** | 22 | 30.99 |
| **No** | 49 | 69.01 |

| **Q19_2_for_trading** | **Frequency** | **Percent** |
| --- | --- | --- |
| **No** | 46 | 64.79 |
| **Trading** | 25 | 35.21 |

| **Q19_3_for_fairs** | **Frequency** | **Percent** |
| --- | --- | --- |
| **Fairs/Field days** | 10 | 14.08 |
| **No** | 61 | 85.92 |

| **Q19_4_for_slaughter** | **Frequency** | **Percent** |
| --- | --- | --- |
| **No** | 33 | 46.48 |
| **Slaughter** | 38 | 53.52 |

| **Q19_5_for_grazing** | **Frequency** | **Percent** |
| --- | --- | --- |
| **Access to feed** | 1 | 1.41 |
| **No** | 70 | 98.59 |

| **Q19_6_for_Swill** | **Frequency** | **Percent** |
| --- | --- | --- |
| **No** | 71 | 100.00 |

| **Q19_7_to_feed** | **Frequency** | **Percent** |
| --- | --- | --- |
| **No** | 71 | 100.00 |

| **Q19_8_not_move** | **Frequency** | **Percent** |
| --- | --- | --- |
| **I do not move pigs** | 22 | 30.99 |
| **No** | 49 | 69.01 |

| **Q20_move_freq** | **Frequency** | **Percent** |
| --- | --- | --- |
| **2 times a year** | 16 | 22.54 |
| **4 times a year** | 16 | 22.54 |
| **I don't move them** | 19 | 26.76 |
| **Monthly** | 10 | 14.08 |
| **Once a year** | 3 | 4.23 |
| **Weekly** | 4 | 5.63 |
| **na** | 3 | 4.23 |

| **Q21_share_vehicle** | **Frequency** | **Percent** |
| --- | --- | --- |
| **No** | 56 | 78.87 |
| **Yes** | 15 | 21.13 |
| **Q22_Share_equipment** | Frequency | Percent |
| **No** | 54 | 76.06 |
| **Yes** | 17 | 23.94 |

| **Q23_1_floor_concrete** | **Frequency** | **Percent** |
| --- | --- | --- |
| **Concrete** | 58 | 81.69 |
| **No** | 13 | 18.31 |

| **Q23_2_floor_dirt** | **Frequency** | **Percent** |
| --- | --- | --- |
| **Dirt** | 20 | 28.17 |
| **No** | 51 | 71.83 |

| **Q23_3_floor_sand** | **Frequency** | **Percent** |
| --- | --- | --- |
| **No** | 69 | 97.18 |
| **Sand** | 2 | 2.82 |

| **Q24_confinement** | **Frequency** | **Percent** |
| --- | --- | --- |
| **Frequency Missing = 1** | | |
| **Always** | 66 | 94.29 |
| **Never** | 2 | 2.86 |
| **Only at night** | 2 | 2.86 |
| **Q25_1_home_consumption** | Frequency | Percent |
| **Home consumption** | 33 | 46.48 |
| **No** | 38 | 53.52 |

| **Q25_2_market** | **Frequency** | **Percent** |
| --- | --- | --- |
| **No** | 25 | 35.21 |
| **Sold in the market** | 46 | 64.79 |

| **Q25_3_sold_farmers** | **Frequency** | **Percent** |
| --- | --- | --- |
| **No** | 49 | 69.01 |
| **Sold to other farmers** | 22 | 30.99 |

| **Q25_4_sold_slaughterhouse** | **Frequency** | **Percent** |
| --- | --- | --- |
| **No** | 44 | 61.97 |
| **Sold to slaughterhouse** | 27 | 38.03 |

| **Q25_5_breeding** | **Frequency** | **Percent** |
| --- | --- | --- |
| **Breeding only** | 4 | 5.63 |
| **No** | 67 | 94.37 |

Q26

| **Q26 monitor** | **Frequency** | **Percent** |
| --- | --- | --- |
| **Extension officer** | 1 | 1.41 |
| **Extension officer and veterinarian** | 1 | 1.41 |
| **Family** | 1 | 1.41 |
| **Farmer** | 24 | 33.80 |
| **Farmer / Worker** | 1 | 1.41 |
| **Farmer, extension officer** | 1 | 1.41 |
| **Farmer, worker, extension officer** | 1 | 1.41 |
| **Father / Uncle** | 1 | 1.41 |
| **Gov/Private Vet** | 1 | 1.41 |
| **Me (farmer) and my father** | 1 | 1.41 |
| **Me [farmer] and my family** | 1 | 1.41 |
| **Owner** | 2 | 2.82 |
| **VPH officer** | 1 | 1.41 |
| **VPH officer, extension officer** | 1 | 1.41 |
| **Veterinarian** | 16 | 22.54 |
| **Veterinary /extension officer** | 1 | 1.41 |
| **Veterinary Unit / Ross University** | 1 | 1.41 |
| **Veterinary office** | 1 | 1.41 |
| **Veterinary officers / extension officers** | 1 | 1.41 |
| **Veterinary services** | 2 | 2.82 |
| **Veterinary unit** | 2 | 2.82 |
| **Workers** | 1 | 1.41 |
| **agric officer, animal health aid officer** | 1 | 1.41 |
| **animal health personnel** | 1 | 1.41 |
| **everyone in the family** | 1 | 1.41 |
| **extension officer and farmer** | 1 | 1.41 |
| **extension officerand veterinarian** | 1 | 1.41 |
| **local vet** | 1 | 1.41 |
| **self (technical farmer/retired VPH officer)** | 1 | 1.41 |
| **the farmer / owner** | 1 | 1.41 |

Q27

| **Q27 what if sick** | | |
| --- | --- | --- |
| **Q27 what if sick** | **Frequency** | **Percent** |
| **-** | 1 | 1.41 |
| **Cal vet services** | 1 | 1.41 |
| **Call Animal doctor** | 1 | 1.41 |
| **Call VPH Officer** | 1 | 1.41 |
| **Call Vet** | 2 | 2.82 |
| **Call the vet** | 3 | 4.23 |
| **Call the veterinary clinic** | 1 | 1.41 |
| **Call vet** | 4 | 5.63 |
| **Call vet / Check myself** | 1 | 1.41 |
| **Call veterinarian** | 1 | 1.41 |
| **Contact the Vet Sharon** | 1 | 1.41 |
| **Contact the vet** | 1 | 1.41 |
| **Do my own thing at times** | 1 | 1.41 |
| **Move them from the rest** | 1 | 1.41 |
| **Separate them** | 1 | 1.41 |
| **Treat them** | 1 | 1.41 |
| **Vet** | 4 | 5.63 |
| **ask another farmer** | 1 | 1.41 |
| **cal vet services** | 1 | 1.41 |
| **call a vet** | 2 | 2.82 |
| **call a vet / extension officer** | 1 | 1.41 |
| **call a vet officer / extension officer** | 1 | 1.41 |
| **call a vet, extension officer** | 1 | 1.41 |
| **call extension officer / vet** | 1 | 1.41 |
| **call for assistance** | 1 | 1.41 |
| **call government vets** | 1 | 1.41 |
| **call ross or vet** | 1 | 1.41 |
| **call the vet** | 5 | 7.04 |
| **call the vet office** | 1 | 1.41 |
| **call the veterinarian** | 1 | 1.41 |
| **call vet** | 7 | 9.86 |
| **call vet or Ross uni** | 1 | 1.41 |
| **call vet services** | 3 | 4.23 |
| **call vet unit** | 1 | 1.41 |
| **call veterinary services** | 1 | 1.41 |
| **contact ross or local vet** | 1 | 1.41 |
| **contact vet** | 1 | 1.41 |
| **contact vet services** | 1 | 1.41 |
| **contact veterinarian** | 1 | 1.41 |
| **ext officer** | 1 | 1.41 |
| **get medicine** | 1 | 1.41 |
| **government veterinarian** | 1 | 1.41 |
| **isolate/quarantine/Call VPH officer** | 1 | 1.41 |
| **myself** | 1 | 1.41 |
| **ross university & local vet** | 1 | 1.41 |
| **self care** | 1 | 1.41 |
| **treat them** | 1 | 1.41 |
| **vet** | 1 | 1.41 |
| **vet/ext officer** | 1 | 1.41 |

| **Q28_1_records_yn** | **Frequency** | **Percent** |
| --- | --- | --- |
| **No** | 35 | 49.30 |
| **Yes** | 36 | 50.70 |

| **Q28_2_handwritten** | **Frequency** | **Percent** |
| --- | --- | --- |
| **No** | 40 | 56.34 |
| **Yes - handwritten** | 31 | 43.66 |

| **Q28_3_digital** | **Frequency** | **Percent** |
| --- | --- | --- |
| **No** | 56 | 78.87 |
| **Yes - digital** | 15 | 21.13 |

| **Q29_1_disease** | **Frequency** | **Percent** |
| --- | --- | --- |
| **Disease** | 23 | 32.39 |
| **No** | 42 | 59.15 |
| **na** | 6 | 8.45 |

| **Q29_2_predators** | **Frequency** | **Percent** |
| --- | --- | --- |
| **No** | 64 | 90.14 |
| **Predators** | 1 | 1.41 |
| **na** | 6 | 8.45 |

| **Q29_3_theft** | **Frequency** | **Percent** |
| --- | --- | --- |
| **No** | 56 | 78.87 |
| **Theft** | 9 | 12.68 |
| **na** | 6 | 8.45 |

| **Q29_4_accidents** | **Frequency** | **Percent** |
| --- | --- | --- |
| **Accidents** | 14 | 19.72 |
| **No** | 51 | 71.83 |
| **na** | 6 | 8.45 |

| **Q29_5_idk** | **Frequency** | **Percent** |
| --- | --- | --- |
| **I don't know** | 27 | 38.03 |
| **No** | 38 | 53.52 |
| **na** | 6 | 8.45 |

| **Q30_selling_sick** | **Frequency** | **Percent** |
| --- | --- | --- |
| **Frequency Missing = 1** | | |
| **No** | 68 | 97.14 |
| **Yes** | 2 | 2.86 |

Q31

| **Q31 whom sold** | | |
| --- | --- | --- |
| **Q31 whom sold** | **Frequency** | **Percent** |
| **-** | 48 | 67.61 |
| **Nobody** | 1 | 1.41 |
| **na** | 12 | 16.90 |
| **never, abattoir** | 1 | 1.41 |
| **no one** | 5 | 7.04 |
| **nobody** | 4 | 5.63 |

| **Q32_price** | **Frequency** | **Percent** |
| --- | --- | --- |
| **Higher price** | 1 | 1.41 |
| **I have never sold a sick pig** | 64 | 90.14 |
| **na** | 6 | 8.45 |

| **Q33_what_after** | **Frequency** | **Percent** |
| --- | --- | --- |
| **I don't know** | 44 | 61.97 |
| **It died** | 6 | 8.45 |
| **It was sent to the abattoir** | 1 | 1.41 |
| **na** | 20 | 28.17 |

| **Q34_know_ASF** | **Frequency** | **Percent** |
| --- | --- | --- |
| **No** | 50 | 70.42 |
| **Yes** | 21 | 29.58 |

| **Q35_ASF_Outbreak** | **Frequency** | **Percent** |
| --- | --- | --- |
| **I don't know** | 16 | 22.54 |
| **No** | 37 | 52.11 |
| **Yes** | 18 | 25.35 |

| **Q36_1_fever** | **Frequency** | **Percent** |
| --- | --- | --- |
| **Fever** | 28 | 39.44 |
| **No** | 5 | 7.04 |
| **na** | 38 | 53.52 |

| **Q36_2_highmortality** | **Frequency** | **Percent** |
| --- | --- | --- |
| **High mortality** | 16 | 22.54 |
| **No** | 17 | 23.94 |
| **na** | 38 | 53.52 |

| **Q36_3_coughing** | **Frequency** | **Percent** |
| --- | --- | --- |
| **Coughing** | 6 | 8.45 |
| **No** | 27 | 38.03 |
| **na** | 38 | 53.52 |

| **Q36_4_diarrhea** | **Frequency** | **Percent** |
| --- | --- | --- |
| **Diarrhea** | 11 | 15.49 |
| **No** | 22 | 30.99 |
| **na** | 38 | 53.52 |

| **Q36_5_vomiting** | **Frequency** | **Percent** |
| --- | --- | --- |
| **No** | 20 | 28.17 |
| **Vomiting** | 13 | 18.31 |
| **na** | 38 | 53.52 |

| **Q36_6_bloodydiarrhea** | **Frequency** | **Percent** |
| --- | --- | --- |
| **Bloody diarrhea** | 3 | 4.23 |
| **No** | 30 | 42.25 |
| **na** | 38 | 53.52 |

| **Q36_7_reducedeating** | **Frequency** | **Percent** |
| --- | --- | --- |
| **No** | 20 | 28.17 |
| **Reduced eating** | 13 | 18.31 |
| **na** | 38 | 53.52 |

| **Q36_8_jointswell** | **Frequency** | **Percent** |
| --- | --- | --- |
| **Joint swelling** | 3 | 4.23 |
| **No** | 30 | 42.25 |
| **na** | 38 | 53.52 |

| **Q36_9_lethargy** | **Frequency** | **Percent** |
| --- | --- | --- |
| **Lethargy** | 1 | 1.41 |
| **No** | 32 | 45.07 |
| **na** | 38 | 53.52 |

| **Q37_1_fever** | **Frequency** | **Percent** |
| --- | --- | --- |
| **Fever** | 34 | 47.89 |
| **No** | 20 | 28.17 |
| **na** | 17 | 23.94 |

| **Q37_2_highmortality** | **Frequency** | **Percent** |
| --- | --- | --- |
| **High mortality** | 16 | 22.54 |
| **No** | 38 | 53.52 |
| **na** | 17 | 23.94 |

| **Q37_3_coughing** | **Frequency** | **Percent** |
| --- | --- | --- |
| **Coughing** | 10 | 14.08 |
| **No** | 44 | 61.97 |
| **na** | 17 | 23.94 |

| **Q37_4_diarrhea** | **Frequency** | **Percent** |
| --- | --- | --- |
| **Diarrhea** | 13 | 18.31 |
| **No** | 41 | 57.75 |
| **na** | 17 | 23.94 |

| **Q37_5_vomiting** | **Frequency** | **Percent** |
| --- | --- | --- |
| **No** | 39 | 54.93 |
| **Vomiting** | 15 | 21.13 |
| **na** | 17 | 23.94 |

| **Q37_6_bloodydiarrhea** | **Frequency** | **Percent** |
| --- | --- | --- |
| **Bloody diarrhea** | 5 | 7.04 |
| **No** | 49 | 69.01 |
| **na** | 17 | 23.94 |

| **Q37_7_reducedeating** | **Frequency** | **Percent** |
| --- | --- | --- |
| **No** | 31 | 43.66 |
| **Reduced eating** | 23 | 32.39 |
| **na** | 17 | 23.94 |

| **Q37_8_jointswell** | **Frequency** | **Percent** |
| --- | --- | --- |
| **Joint swelling** | 4 | 5.63 |
| **No** | 50 | 70.42 |
| **na** | 17 | 23.94 |

| **Q37_9_lethargy** | **Frequency** | **Percent** |
| --- | --- | --- |
| **Lethargy** | 8 | 11.27 |
| **No** | 46 | 64.79 |
| **na** | 17 | 23.94 |

| **Q37_10_bloodurine** | **Frequency** | **Percent** |
| --- | --- | --- |
| **Blood in urine** | 2 | 2.82 |
| **No** | 52 | 73.24 |
| **na** | 17 | 23.94 |

| **Q37_11_hemorraghes** | **Frequency** | **Percent** |
| --- | --- | --- |
| **Hemorrhages** | 4 | 5.63 |
| **No** | 50 | 70.42 |
| **na** | 17 | 23.94 |

| **Q37_12_othersigns** | **Frequency** | **Percent** |
| --- | --- | --- |
| **No** | 51 | 71.83 |
| **Other signs** | 3 | 4.23 |
| **na** | 17 | 23.94 |

| **Q38 other name** | | |
| --- | --- | --- |
| **Q38 other name** | **Frequency** | **Percent** |
| **-** | 34 | 47.89 |
| **?** | 1 | 1.41 |
| **Have no idea** | 1 | 1.41 |
| **I don know** | 1 | 1.41 |
| **I dont know** | 2 | 2.82 |
| **No** | 7 | 9.86 |
| **Pig Fever** | 1 | 1.41 |
| **i dont know** | 1 | 1.41 |
| **idk** | 1 | 1.41 |
| **na** | 6 | 8.45 |
| **no** | 15 | 21.13 |
| **nopes** | 1 | 1.41 |

| **Q39_1_callvet** | **Frequency** | **Percent** |
| --- | --- | --- |
| **No** | 6 | 8.45 |
| **Veterinarian** | 63 | 88.73 |
| **na** | 2 | 2.82 |

| **Q39_2_callextoff** | | |
| --- | --- | --- |
| **Q39_2_callextoff** | **Frequency** | **Percent** |
| **Extension officer** | 46 | 64.79 |
| **No** | 23 | 32.39 |
| **na** | 2 | 2.82 |

| **Q39_3_callotherfarmers** | **Frequency** | **Percent** |
| --- | --- | --- |
| **No** | 58 | 81.69 |
| **Other farmers** | 11 | 15.49 |
| **na** | 2 | 2.82 |

| **Q39_4_callfamily** | **Frequency** | **Percent** |
| --- | --- | --- |
| **Family members** | 2 | 2.82 |
| **No** | 67 | 94.37 |
| **na** | 2 | 2.82 |

| **Q39_5_callnoone** | **Frequency** | **Percent** |
| --- | --- | --- |
| **No** | 68 | 95.77 |
| **No one** | 1 | 1.41 |
| **na** | 2 | 2.82 |

| **Q40_practice_change** | | |
| --- | --- | --- |
| **Q40_practice_change** | **Frequency** | **Percent** |
| **-** | 12 | 16.90 |
| **Away from other pigs Treatment and monitor carefully More grass** | 1 | 1.41 |
| **Biosecurity** | 2 | 2.82 |
| **Biosecurity, movements, treatment, weed control** | 1 | 1.41 |
| **Biosecurity, movements, treatments** | 2 | 2.82 |
| **Biosecurity/movement** | 1 | 1.41 |
| **Change of feed, pen cleaning, and checking** | 1 | 1.41 |
| **Change treatment** | 1 | 1.41 |
| **Cleaning, Quarantine** | 1 | 1.41 |
| **Culling** | 8 | 11.27 |
| **Food change (less oil products) Provide fresh grass / hay** | 1 | 1.41 |
| **I would look at food** | 1 | 1.41 |
| **Movement/Biosecurity** | 1 | 1.41 |
| **Movements** | 1 | 1.41 |
| **Sanitation protocols** | 1 | 1.41 |
| **Start move sick from healthy Start kill to avoid spread (culling)** | 1 | 1.41 |
| **Treatment** | 6 | 8.45 |
| **Treatment if possible** | 1 | 1.41 |
| **Treatment what vet says** | 1 | 1.41 |
| **Try to keep separated from the rest of the flock** | 1 | 1.41 |
| **Yes, by separating them** | 1 | 1.41 |
| **biosecurity** | 3 | 4.23 |
| **biosecurity and movements** | 2 | 2.82 |
| **biosecurity, movement, cull, treatment** | 1 | 1.41 |
| **medicine (worm medicine)** | 1 | 1.41 |
| **move them to an area** | 1 | 1.41 |
| **movement, biosecurity** | 1 | 1.41 |
| **movements** | 2 | 2.82 |
| **movements and treatments** | 1 | 1.41 |
| **no change** | 1 | 1.41 |
| **no movement of pigs and people on farms** | 1 | 1.41 |
| **treat animals** | 1 | 1.41 |
| **treatment** | 3 | 4.23 |
| **treatment & move Isolate Biosecurity** | 1 | 1.41 |
| **treatment and biosecurity** | 1 | 1.41 |
| **treatment and movements** | 3 | 4.23 |
| **treatment for pigs** | 1 | 1.41 |
| **treatment, biosecurity, movement** | 1 | 1.41 |

| **Q41_move_if_sick** | **Frequency** | **Percent** |
| --- | --- | --- |
| **Frequency Missing = 1** | | |
| **No** | 61 | 87.14 |
| **Yes** | 9 | 12.86 |

| **Q41a_1_not_move_sick_pigs** | **Frequency** | **Percent** |
| --- | --- | --- |
| **I would not move sick pigs** | 44 | 61.97 |
| **No** | 8 | 11.27 |
| **na** | 19 | 26.76 |

| **Q41a_2_to_veterinarian** | **Frequency** | **Percent** |
| --- | --- | --- |
| **No** | 45 | 63.38 |
| **To the veterinarian** | 7 | 9.86 |
| **na** | 19 | 26.76 |

| **Q41a_3_to_abattoir** | **Frequency** | **Percent** |
| --- | --- | --- |
| **Abattoir** | 2 | 2.82 |
| **No** | 50 | 70.42 |
| **na** | 19 | 26.76 |

| **Q41a_4_to_otherpen** | **Frequency** | **Percent** |
| --- | --- | --- |
| **No** | 51 | 71.83 |
| **To another pen** | 1 | 1.41 |
| **na** | 19 | 26.76 |

| **Q42_people_access** | **Frequency** | **Percent** |
| --- | --- | --- |
| **No** | 11 | 15.49 |
| **Yes** | 58 | 81.69 |
| **na** | 2 | 2.82 |

Q43

| **Q43 not report** | | |
| --- | --- | --- |
| **Q43 not report** | **Frequency** | **Percent** |
| **-** | 30 | 42.25 |
| **?** | 1 | 1.41 |
| **But why shouldn't I?** | 1 | 1.41 |
| **I dont know I should not** | 1 | 1.41 |
| **I have never heard that** | 1 | 1.41 |
| **I have no idea** | 2 | 2.82 |
| **Might lose all the pigs based on advise from vet** | 1 | 1.41 |
| **Never** | 1 | 1.41 |
| **Never heard of that** | 1 | 1.41 |
| **Never heard that** | 2 | 2.82 |
| **No** | 1 | 1.41 |
| **No knowledge** | 1 | 1.41 |
| **No reason** | 1 | 1.41 |
| **No, I would always report events of high mortality to extension officer /VPH officer** | 1 | 1.41 |
| **Nopes** | 2 | 2.82 |
| **Reporting will be done to vet/ext officer** | 1 | 1.41 |
| **i should always report a mortality matter** | 1 | 1.41 |
| **i think it should always be reported** | 1 | 1.41 |
| **i will always report it to the vet** | 1 | 1.41 |
| **na** | 1 | 1.41 |
| **no** | 13 | 18.31 |
| **no answer** | 1 | 1.41 |
| **no knowledge** | 1 | 1.41 |
| **no, I have no idea why** | 1 | 1.41 |
| **no, i dont have a clue** | 1 | 1.41 |
| **no, i have no idea why** | 1 | 1.41 |
| **not important** | 1 | 1.41 |

Q44

| **Q44 why not** | | |
| --- | --- | --- |
| **Q44 why not** | **Frequency** | **Percent** |
| **-** | 10 | 14.08 |
| **Absence of cases on the island** | 3 | 4.23 |
| **Absence of cases on the island & there is a low likelihood of it on my farm** | 1 | 1.41 |
| **Absence of cases on the island, lack of information** | 1 | 1.41 |
| **Because its not on island** | 1 | 1.41 |
| **Depend on the signs** | 1 | 1.41 |
| **Do not know** | 1 | 1.41 |
| **I do not know much about ASF** | 1 | 1.41 |
| **I don't know about the disease** | 1 | 1.41 |
| **I don't think it's on the island** | 1 | 1.41 |
| **I just hope it never reaches here** | 1 | 1.41 |
| **IDK** | 1 | 1.41 |
| **Its not here** | 3 | 4.23 |
| **Lack of communication** | 1 | 1.41 |
| **Lack of communication from the Vet** | 1 | 1.41 |
| **Lack of information** | 2 | 2.82 |
| **Lack of information about it** | 1 | 1.41 |
| **Lack of information from agricultural department** | 1 | 1.41 |
| **Lack of information from vets, government, media, farmers.** | 1 | 1.41 |
| **Lack of information on suid diseases** | 1 | 1.41 |
| **Lack of information on the island** | 1 | 1.41 |
| **Lack of information or communication about it from government or the press/media** | 1 | 1.41 |
| **Lack of information or communication about it from other farmers** | 1 | 1.41 |
| **Lack of knowledge** | 1 | 1.41 |
| **No** | 1 | 1.41 |
| **No information** | 1 | 1.41 |
| **No information is surfaced** | 1 | 1.41 |
| **No knowledge** | 1 | 1.41 |
| **Nor information nor communication** | 1 | 1.41 |
| **Not much information on it** | 1 | 1.41 |
| **because i dont know information on the disease** | 1 | 1.41 |
| **don't know about symptoms** | 1 | 1.41 |
| **don't know what is ASF** | 1 | 1.41 |
| **dont know** | 1 | 1.41 |
| **lack of cases of ASF** | 1 | 1.41 |
| **lack of communication** | 3 | 4.23 |
| **lack of communication about it from vet** | 1 | 1.41 |
| **lack of communication and information** | 1 | 1.41 |
| **lack of communication from the vet** | 1 | 1.41 |
| **lack of communication with the government** | 1 | 1.41 |
| **lack of information** | 7 | 9.86 |
| **lack of information from the govm't** | 1 | 1.41 |
| **lack of interaction** | 1 | 1.41 |
| **lack of knowledge** | 1 | 1.41 |
| **na** | 2 | 2.82 |
| **no information** | 1 | 1.41 |
| **no knowledge** | 1 | 1.41 |
| **no knowledge of disease** | 1 | 1.41 |

| **Q45_transmission** | **Frequency** | **Percent** |
| --- | --- | --- |
| **I am not informed at all** | 34 | 47.89 |
| **I am somewhat well informed** | 14 | 19.72 |
| **I am well informed** | 6 | 8.45 |
| **I lack some information** | 17 | 23.94 |

| **Q46_1_pigs** | **Frequency** | **Percent** |
| --- | --- | --- |
| **Frequency Missing = 1** | | |
| **Contact with domestic pigs** | 46 | 65.71 |
| **No** | 12 | 17.14 |
| **na** | 12 | 17.14 |

| **Q46_2_feralpigs** | **Frequency** | **Percent** |
| --- | --- | --- |
| **Contact with feral pigs** | 32 | 45.07 |
| **No** | 27 | 38.03 |
| **na** | 12 | 16.90 |

| **Q46_3_food** | **Frequency** | **Percent** |
| --- | --- | --- |
| **Contact with contaminated food or feed** | 41 | 57.75 |
| **No** | 18 | 25.35 |
| **na** | 12 | 16.90 |

| **Q46_4_swill** | **Frequency** | **Percent** |
| --- | --- | --- |
| **Frequency Missing = 1** | | |
| **Contact with swill** | 23 | 32.86 |
| **No** | 35 | 50.00 |
| **na** | 12 | 17.14 |

| **Q46_5_objects** | **Frequency** | **Percent** |
| --- | --- | --- |
| **Frequency Missing = 1** | | |
| **Contact with contaminated objects** | 28 | 40.00 |
| **No** | 30 | 42.86 |
| **na** | 12 | 17.14 |

| **Q46_6_ticks** | **Frequency** | **Percent** |
| --- | --- | --- |
| **Frequency Missing = 1** | | |
| **No** | 35 | 50.00 |
| **Tick bites (some species)** | 23 | 32.86 |
| **na** | 12 | 17.14 |

| **Q46_7_vehicles** | **Frequency** | **Percent** |
| --- | --- | --- |
| **Frequency Missing = 1** | | |
| **Contact with contaminated vehicles** | 28 | 40.00 |
| **No** | 30 | 42.86 |
| **na** | 12 | 17.14 |

| **Q47 visitors** | | |
| --- | --- | --- |
| **Q47 visitors** | **Frequency** | **Percent** |
| **-** | 12 | 16.90 |
| **2 persons visit my farm. No special clothing** | 1 | 1.41 |
| **3 people visit my farm. I limit vehicles on the farm. i don't ask about clothing, etc.** | 1 | 1.41 |
| **5 persons visit the farm I don't ask them about clothing, vehicles, etc.** | 1 | 1.41 |
| **6 persons visit the farm. No biosecurity measures** | 1 | 1.41 |
| **Hardly any visitors** | 1 | 1.41 |
| **Just Vet and extension officer** | 1 | 1.41 |
| **Just me alone** | 1 | 1.41 |
| **Just the vet** | 1 | 1.41 |
| **Just the workers contact with the pigs and the veterinary unit** | 1 | 1.41 |
| **No** | 8 | 11.27 |
| **No biosecurity measures** | 1 | 1.41 |
| **No visitors** | 1 | 1.41 |
| **No visitors allowed** | 1 | 1.41 |
| **No visitors, only the Vet** | 1 | 1.41 |
| **No, not really** | 1 | 1.41 |
| **Not much visitors and they are asked to do nothing** | 1 | 1.41 |
| **Only the E.O. and Vet** | 1 | 1.41 |
| **Only the vet** | 1 | 1.41 |
| **Only worker is allowed on farm** | 1 | 1.41 |
| **Other farmers** | 1 | 1.41 |
| **Sometimes** | 1 | 1.41 |
| **Sometimes (1 time / month)** | 1 | 1.41 |
| **Use my footbath before coming on the farm** | 1 | 1.41 |
| **Vet and extension officer** | 1 | 1.41 |
| **Vet unit** | 1 | 1.41 |
| **Wash boots before entering the farm** | 1 | 1.41 |
| **change of clothes** | 1 | 1.41 |
| **clean boots and vehicle wheels before coming to farm** | 1 | 1.41 |
| **clean wheels of vehicles and wear clean clothing** | 1 | 1.41 |
| **no** | 10 | 14.08 |
| **no visitors** | 2 | 2.82 |
| **no, no, no** | 1 | 1.41 |
| **no, they dont come into my premises** | 1 | 1.41 |
| **no, they state at the perimeter and state their business** | 1 | 1.41 |
| **no, we don't allow for people in our farm** | 1 | 1.41 |
| **no, we dont allow people on the farms** | 1 | 1.41 |
| **not really** | 1 | 1.41 |
| **sometimes** | 2 | 2.82 |
| **yes (especially students)** | 1 | 1.41 |
| **yes, but none come into contact with my pigs** | 1 | 1.41 |
| **yes, many people, buyers** | 1 | 1.41 |

| **Q48 disposal** | | |
| --- | --- | --- |
| **Q48 disposal** | **Frequency** | **Percent** |
| **Frequency Missing = 1** | | |
| **Burn** | 3 | 4.29 |
| **Burn,Throw away** | 1 | 1.43 |
| **Bury** | 30 | 42.86 |
| **Bury,Burn** | 10 | 14.29 |
| **Bury,Burn,Throw away** | 2 | 2.86 |
| **Bury,Landfill** | 2 | 2.86 |
| **Bury,Throw away** | 3 | 4.29 |
| **Landfill** | 3 | 4.29 |
| **Throw away** | 15 | 21.43 |
| **Throw away,Landfill** | 1 | 1.43 |

| **Q48_1_bury** | **Frequency** | **Percent** |
| --- | --- | --- |
| **Frequency Missing = 1** | | |
| **Bury** | 47 | 67.14 |
| **No** | 23 | 32.86 |

| **Q48_2_burn** | **Frequency** | **Percent** |
| --- | --- | --- |
| **Frequency Missing = 1** | | |
| **Burn** | 16 | 22.86 |
| **No** | 54 | 77.14 |

| **Q48_3_throw** | **Frequency** | **Percent** |
| --- | --- | --- |
| **Frequency Missing = 1** | | |
| **No** | 48 | 68.57 |
| **Throw away** | 22 | 31.43 |

| **Q48_4_landfill** | **Frequency** | **Percent** |
| --- | --- | --- |
| **Frequency Missing = 1** | | |
| **Landfill** | 6 | 8.57 |
| **No** | 64 | 91.43 |

| **Q49_feral_concern** | **Frequency** | **Percent** |
| --- | --- | --- |
| **Frequency Missing = 3** | | |
| **No** | 42 | 61.76 |
| **Yes, because they carry diseases** | 17 | 25.00 |
| **Yes, because they carry diseases,Yes, because they breed with my sows** | 1 | 1.47 |
| **Yes, because they carry diseases,Yes, because they destroy my crops** | 4 | 5.88 |
| **Yes, because they carry diseases,Yes, because they eat my pigs feed,Yes, because they fight my animals,Yes, because they destroy my crops** | 1 | 1.47 |
| **Yes, because they eat my pigs feed** | 2 | 2.94 |
| **Yes, because they eat my pigs feed,Yes, because they destroy my crops** | 1 | 1.47 |

| **Q50_pig_contact** | **Frequency** | **Percent** |
| --- | --- | --- |
| **Never** | 62 | 87.32 |
| **Sometimes** | 6 | 8.45 |
| **Yes** | 2 | 2.82 |
| **na** | 1 | 1.41 |

| **Q51_interacting** | **Frequency** | **Percent** |
| --- | --- | --- |
| **-** | 15 | 21.13 |
| **No** | 52 | 73.24 |
| **No wild pigs on this island** | 1 | 1.41 |
| **We dont have those** | 1 | 1.41 |
| **na** | 2 | 2.82 |

| **Q52_Pig_dis** | **Frequency** | **Percent** |
| --- | --- | --- |
| **Frequency Missing = 1** | | |
| **Internet - Social Media** | 4 | 5.71 |
| **Internet - Social Media,Other farmers** | 1 | 1.43 |
| **Internet - Social Media,Veterinarian/Extension officer** | 10 | 14.29 |
| **Internet - Social Media,Veterinarian/Extension officer,Other farmers** | 2 | 2.86 |
| **Internet - Social Media,Veterinarian/Extension officer,Other farmers,Friends** | 2 | 2.86 |
| **Other farmers** | 2 | 2.86 |
| **Radio** | 2 | 2.86 |
| **TV,Internet - Social Media** | 3 | 4.29 |
| **TV,Internet - Social Media,Veterinarian/Extension officer** | 5 | 7.14 |
| **TV,Internet - Social Media,Veterinarian/Extension officer,Other farmers** | 3 | 4.29 |
| **TV,Internet - Social Media,Veterinarian/Extension officer,Other farmers,Friends** | 5 | 7.14 |
| **TV,Radio** | 3 | 4.29 |
| **TV,Radio,Internet - Social Media,Veterinarian/Extension officer,Other farmers** | 3 | 4.29 |
| **TV,Radio,Internet - Social Media,Veterinarian/Extension officer,Other farmers,Friends** | 3 | 4.29 |
| **TV,Radio,Internet - Social Media,Veterinarian/Extension officer,Other farmers,Friends,Family** | 1 | 1.43 |
| **TV,Veterinarian/Extension officer** | 1 | 1.43 |
| **Veterinarian/Extension officer** | 14 | 20.00 |
| **Veterinarian/Extension officer,Other farmers** | 4 | 5.71 |
| **Veterinarian/Extension officer,Other farmers,Friends** | 1 | 1.43 |
| **Veterinarian/Extension officer,Other farmers,Friends,Family** | 1 | 1.43 |

Q53

| **Q53 role dis** | | |
| --- | --- | --- |
| **Q53 role dis** | **Frequency** | **Percent** |
| **Frequency Missing = 1** | | |
| **-** | 13 | 18.57 |
| **Being vigilant about my surroundings** | 1 | 1.43 |
| **Biosecurity Observation of stall** | 1 | 1.43 |
| **Biosecurity, enhancing biosecurity** | 1 | 1.43 |
| **Call of a vet (local vet)** | 1 | 1.43 |
| **Call vet to come and treat animals** | 1 | 1.43 |
| **Check ups from the vet** | 1 | 1.43 |
| **Do not sell No farm visit** | 1 | 1.43 |
| **GAP** | 4 | 5.71 |
| **Give animal fresh water** | 1 | 1.43 |
| **Give clean water** | 1 | 1.43 |
| **Good Agricultural Practices** | 1 | 1.43 |
| **Have a clean pen** | 1 | 1.43 |
| **Have monthly visits by vet** | 1 | 1.43 |
| **I don't know** | 1 | 1.43 |
| **Keep farm clean, have vet make regular checks, Keep stray and feral animals away from the farm** | 1 | 1.43 |
| **Keep my animals away from feral animals** | 1 | 1.43 |
| **Keep my pen area clean** | 1 | 1.43 |
| **Keeping pen clear, have pigs checked out by veterinary officer** | 1 | 1.43 |
| **Less people interact with pigs** | 1 | 1.43 |
| **Limited visitors** | 1 | 1.43 |
| **Management practices** | 1 | 1.43 |
| **Monitor animals for Sickness** | 1 | 1.43 |
| **Monitoring pig health & good management practices** | 1 | 1.43 |
| **Monthly check ups by vet** | 1 | 1.43 |
| **No contact with other animals, keep clean** | 1 | 1.43 |
| **Pay attention to the feeding, handling and cleaning of the area were pigs resides** | 1 | 1.43 |
| **Practice self hygiene and been vigilant** | 1 | 1.43 |
| **Prevent food contamination** | 1 | 1.43 |
| **Protect animals** | 1 | 1.43 |
| **Reading more and try to be as clean as possible** | 1 | 1.43 |
| **Sanitation / food baths, etc.** | 1 | 1.43 |
| **Sanitation measures** | 1 | 1.43 |
| **Spread the word Proper sanitation** | 1 | 1.43 |
| **Treat animals regularly** | 1 | 1.43 |
| **Treat sick animals on time** | 1 | 1.43 |
| **Work as clean as possible** | 1 | 1.43 |
| **[closevind] SIC, closing?** | 1 | 1.43 |
| **clean frequently** | 1 | 1.43 |
| **clean housing** | 1 | 1.43 |
| **clean pens regularly** | 1 | 1.43 |
| **control & biosecurity measures** | 1 | 1.43 |
| **give my animals regular check by vet** | 1 | 1.43 |
| **isolate my animals from feral animals** | 1 | 1.43 |
| **isolate my pigs from feral animals** | 1 | 1.43 |
| **keep feral animals from my pemises** | 1 | 1.43 |
| **keep my animals away from wild animals** | 1 | 1.43 |
| **keeping my animals away from wild animals** | 1 | 1.43 |
| **keeping surroundings clean** | 1 | 1.43 |
| **knowledge** | 1 | 1.43 |
| **let the vet check my animals regularly** | 1 | 1.43 |
| **limit entry (gates)** | 1 | 1.43 |
| **prevent feed contamination** | 1 | 1.43 |
| **proper management practices** | 1 | 1.43 |
| **provide clean water** | 1 | 1.43 |

| **Q54_1_S_Closedherd** | **Frequency** | **Percent** |
| --- | --- | --- |
| **No** | 38 | 53.52 |
| **[Segregation] Closed herd** | 32 | 45.07 |
| **na** | 1 | 1.41 |

| **Q54_2_S_Accessrestriction** | **Frequency** | **Percent** |
| --- | --- | --- |
| **No** | 32 | 45.07 |
| **[Segregation] Access restriction** | 38 | 53.52 |
| **na** | 1 | 1.41 |

| **Q54_3_S_Cleandirtyareas** | **Frequency** | **Percent** |
| --- | --- | --- |
| **No** | 23 | 32.39 |
| **[Segregation] Clean/Dirty areas** | 47 | 66.20 |
| **na** | 1 | 1.41 |

| **Q54_4_S_Preventcontact** | **Frequency** | **Percent** |
| --- | --- | --- |
| **No** | 34 | 47.89 |
| **[Segregation] Prevent contact (animals and people)** | 36 | 50.70 |
| **na** | 1 | 1.41 |

| **Q54_5_S_Pestcontrol** | **Frequency** | **Percent** |
| --- | --- | --- |
| **No** | 36 | 50.70 |
| **[Segregation] Pest/Insect control** | 34 | 47.89 |
| **na** | 1 | 1.41 |

| **Q54_6_CD_Facilities** | **Frequency** | **Percent** |
| --- | --- | --- |
| **No** | 20 | 28.17 |
| **[Cleaning & Disinfection] Facilities** | 50 | 70.42 |
| **na** | 1 | 1.41 |

| **Q54_7_CD_Equipment** | **Frequency** | **Percent** |
| --- | --- | --- |
| **No** | 30 | 42.25 |
| **[Cleaning & Disinfection] Equipment** | 40 | 56.34 |
| **na** | 1 | 1.41 |

| **Q54_8_CD_Vehicles** | **Frequency** | **Percent** |
| --- | --- | --- |
| **No** | 35 | 49.30 |
| **[Cleaning & Disinfection] Vehicles** | 35 | 49.30 |
| **na** | 1 | 1.41 |

| **Q55_1_lack_time** | **Frequency** | **Percent** |
| --- | --- | --- |
| **Lack of resources (time)** | 37 | 52.11 |
| **No** | 33 | 46.48 |
| **na** | 1 | 1.41 |

| **Q55_2_lack_money** | **Frequency** | **Percent** |
| --- | --- | --- |
| **Lack of resources (money)** | 50 | 70.42 |
| **No** | 20 | 28.17 |
| **na** | 1 | 1.41 |

| **Q55_3_lack_knowledge** | **Frequency** | **Percent** |
| --- | --- | --- |
| **Lack of resources (knowledge)** | 35 | 49.30 |
| **No** | 35 | 49.30 |
| **na** | 1 | 1.41 |
